# Epigenetic regulation of the tumor microenvironment in lung cancer: mechanism insights and therapeutic prospects

**DOI:** 10.3389/fimmu.2025.1697428

**Published:** 2025-11-28

**Authors:** Maoqin Yang, Xudong Lei, Dexiang Ren, Dakai Qin, Xiaojun Xia

**Affiliations:** 1 Gansu University Of Chinese Medicine, Lanzhou, Gansu, China; 2 Gansu Provincial Cancer Hospital, Lanzhou, Gansu, China

**Keywords:** lung cancer, tumor microenvironment, DNA methylation, histone modification, non-coding RNA

## Abstract

Lung cancer, recognized as one of the most prevalent malignancies with the highest rates of incidence and mortality globally, presents a substantial challenge on a worldwide scale. This challenge is exacerbated by the disease’s difficulty in early detection, a pronounced rate of metastasis, and resistance to treatment, all of which contribute to elevated mortality rates. The tumor microenvironment (TME) plays a critical role in the sustenance and advancement of various solid tumors, including lung cancer. The intricate composition of the TME facilitates tumor proliferation, metastatic spread, and therapeutic resistance by supplying metabolic resources, fostering angiogenesis, and enabling immune evasion. Nonetheless, the regulatory frameworks operating within the TME remain poorly understood. An increasing body of evidence suggests that epigenetic regulation—encompassing mechanisms such as DNA methylation, histone modification, and the action of non-coding RNAs—is pivotal in the initiation and progression of lung cancer. Furthermore, epigenetic modifications significantly influence the functional dynamics of the tumor microenvironment, thereby impacting intercellular interactions and cellular behaviors within the TME, which in turn affects the trajectory of disease progression. This article aims to present the most recent advancements in research concerning the epigenetic regulation of tumor cell interactions with the TME in the context of lung cancer biology. Additionally, it examines the current implications of epigenetic regulation within the tumor microenvironment and its influence on lung cancer behavior. We also investigate the potential relevance and emerging therapeutic avenues presented by epigenetic regulation in the clinical diagnosis and treatment of lung cancer, aspiring to propose novel strategies to address existing treatment challenges.

## 1 Introduction

Lung cancer, recognized as the malignant neoplasm with the highest rates of incidence and mortality globally, poses a significant threat to human health and well-being (1). According to the most recent data on the global cancer burden published by the International Agency for Research on Cancer (IARC), part of the World Health Organization, in 2020, there were approximately 2.2 million newly diagnosed lung cancer cases, with a staggering 1.8 million resulting in death (2). These figures represent 11.4% of all newly diagnosed cancer cases and 18.0% of cancer-related fatalities worldwide. Lung cancer is primarily categorized into two types: non-small cell lung cancer (NSCLC), which constitutes about 85% of cases, and small cell lung cancer (SCLC), accounting for roughly 15% (3, 4). Despite noteworthy advancements in lung cancer treatment modalities over recent years-including surgical intervention, chemotherapy, radiotherapy, targeted therapy, and immunotherapy-the overall five-year survival rate for patients remains at a disappointing 18% (5, 6). This dismal statistic is largely due to challenges associated with the early detection of the disease, the tumors’ high metastatic capabilities, and resistance to currently available therapies (7, 8). Therefore, a comprehensive understanding of the pathophysiology of lung cancer, along with the identification of novel therapeutic targets and strategies, is crucial for enhancing the prognosis of lung cancer patients.

Research has indicated that the tumor microenvironment (TME) is pivotal in tumor progression and maintenance (9, 10). As the immediate environment that supports the survival and development of tumor cells, the TME is a multifaceted structure that includes tumor cells, immune cells, stromal cells, extracellular matrix components, and an array of bioactive molecules (11). Within the cellular constituents of the TME, cancer-associated fibroblasts (CAFs) are notable for their ability to secrete various growth factors and extracellular matrix components, which not only provide essential nutritional support but also enhance the proliferation and migration of tumor cells (12, 13). Macrophages within the TME exhibit heterogeneity, with M2-type macrophages capable of releasing pro-angiogenic factors, such as vascular endothelial growth factor (VEGF), thereby promoting tumor angiogenesis and aiding in the metastasis of tumor cells (14, 15). Furthermore, immune cells present in the TME, including T cells and natural killer (NK) cells, are critical for tumor immune surveillance and the mechanisms of immune evasion. When immune evasion transpires, tumor cells can successfully avoid the host immune system’s attacks, subsequently leading to metastatic spread (16, 17). Lung cancer cells interact with a variety of cells and molecules within the TME, establishing a complex ecosystem. This ecosystem not only creates favorable conditions for the growth and survival of lung cancer cells but also significantly influences their biological behaviors and responses to therapeutic interventions (18, 19).

**Figure 1:**
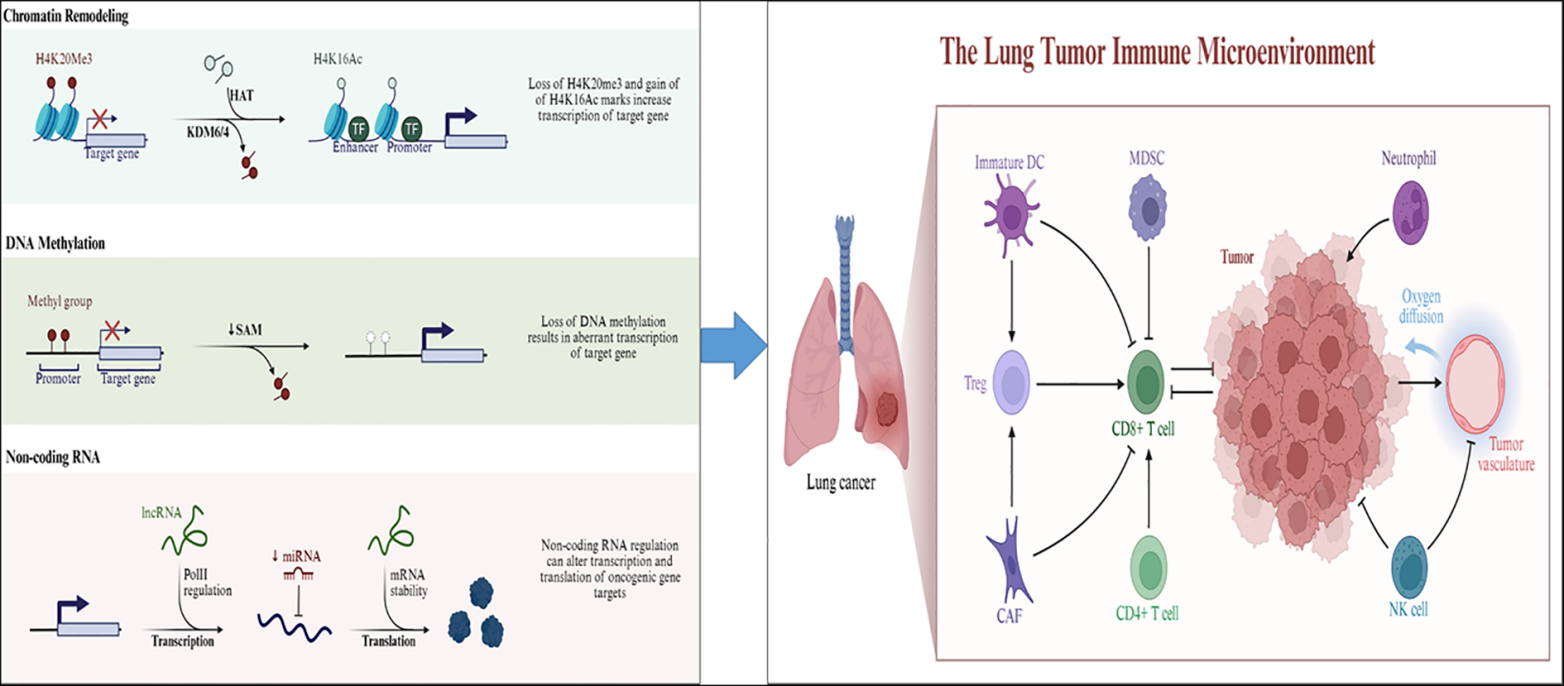
The relationship between epigenetic regulation (DNA methylation, histone modification, non-coding RNA regulation) and the microenvironment of lung cancer. (Left panel) Major epigenetic mechanisms—including chromatin remodeling, DNA methylation, and non-coding RNA regulation—contribute to aberrant gene expression in lung cancer. In chromatin remodeling, loss of the repressive mark H4K20me3 and gain of the active mark H4K16Ac through KDM6/4 and HAT activity enhance chromatin accessibility and transcription of oncogenic target genes. In DNA methylation, reduced S-adenosylmethionine (SAM) levels and DNA hypomethylation lead to abnormal activation of tumor-promoting genes. In non-coding RNA regulation, lncRNAs and miRNAs modulate gene expression by influencing Pol II–mediated transcription, mRNA stability, and translation of oncogenic transcripts.(Right panel) These epigenetic alterations collectively reshape the lung tumor immune microenvironment (TIME). Aberrant gene regulation affects key immune populations, including immature dendritic cells (DCs), myeloid-derived suppressor cells (MDSCs), regulatory T cells (Tregs), cancer-associated fibroblasts (CAFs), CD4^+^/CD8^+^ T cells, NK cells, and neutrophils. Epigenetic reprogramming in tumor and stromal cells impairs antigen presentation, promotes immunosuppression, and alters oxygen diffusion and vascular remodeling, ultimately leading to immune escape and tumor progression.(activating (→) and inhibitory (⟞)).

Epigenetic regulation denotes the mechanisms that modulate gene expression without modifying the underlying DNA sequence. The primary forms of epigenetic regulation encompass DNA methylation, histone modifications, and the influence of non-coding RNAs (ncRNAs) (20–22). Such epigenetic alterations play pivotal roles in both physiological and pathological processes, including organismal growth and development, cellular differentiation, aging, as well as the initiation and advancement of various diseases (23–25). Recent investigations have highlighted the significant involvement of epigenetic mechanisms in the development of lung cancer. These mechanisms can affect the progression of lung cancer by altering cellular functions and facilitating intercellular communication within the TME (26, 27) (**Figure 1**). Nonetheless, a thorough comprehension of the epigenetic mechanisms that contribute to TME modifications remains elusive. This article aims to discuss the most recent findings regarding epigenetic events that influence interactions with the tumor microenvironment in the context of lung cancer biology. Additionally, we will examine their potential clinical implications, including the identification of epigenetic biomarkers and therapeutic strategies.

## 2 Overview of the microenvironment of lung cancer

**Figure 2:**
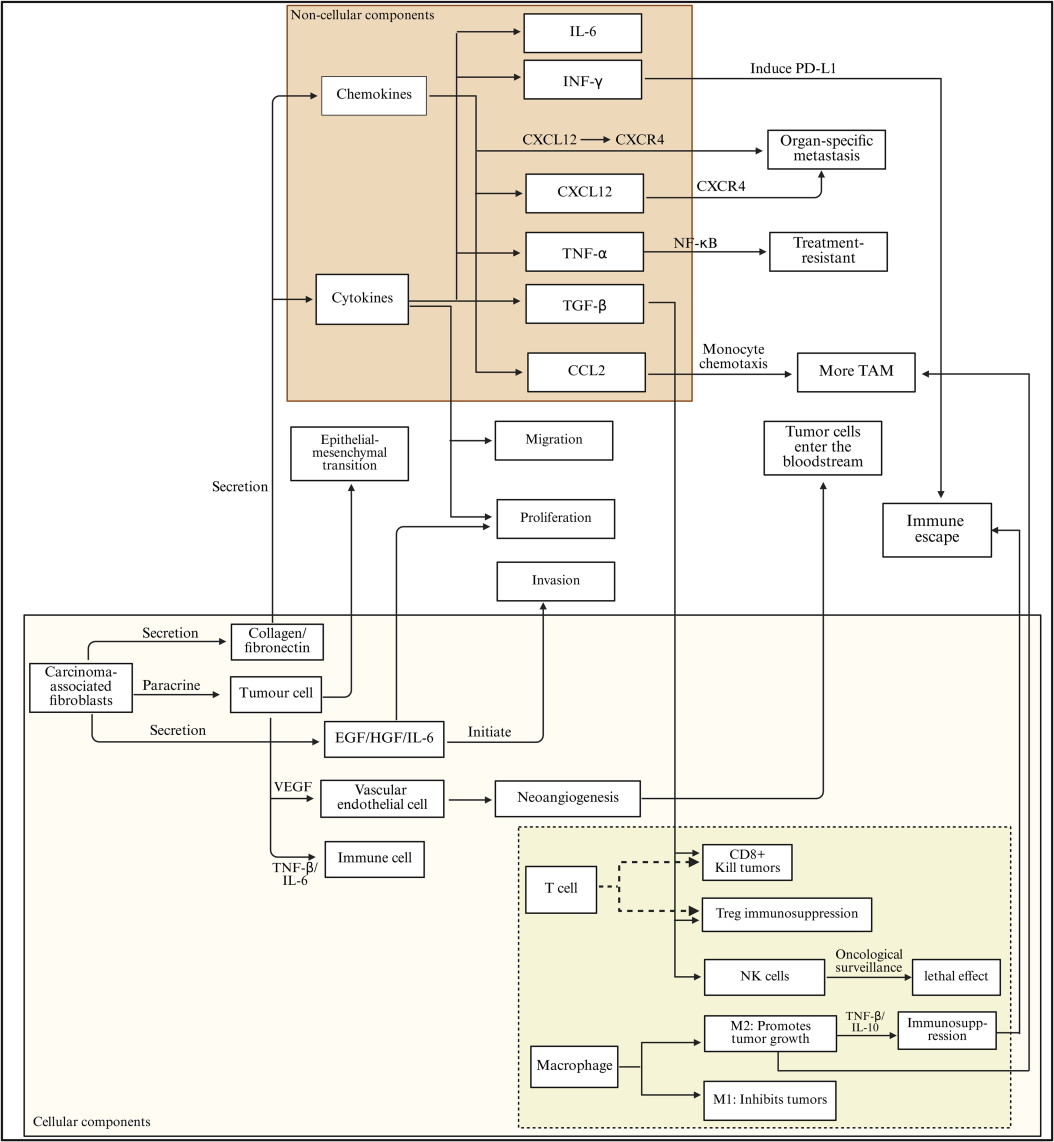
Cytokine–chemokine network and cellular crosstalk driving immune escape in the lung tumor microenvironment. Cytokines and chemokines within the lung tumor microenvironment orchestrate a complex signaling network that promotes tumor progression and immune evasion. Key mediators such as IL-6, IFN-γ, CXCL12, TNF-α, TGF-β, and CCL2 regulate epithelial–mesenchymal transition (EMT), proliferation, migration, invasion, and neoangiogenesis. Through activation of CXCL12/CXCR4 and NF-κB signaling, these factors induce PD-L1 expression, enhance monocyte chemotaxis, and drive the recruitment of tumor-associated macrophages (TAMs), contributing to metastasis and treatment resistance. In parallel, carcinoma-associated fibroblasts secrete EGF, HGF, and IL-6, which stimulate tumor and endothelial cells to initiate vascular remodeling and immune suppression. Within the immune compartment, CD8^+^ T cells and NK cells mediate cytotoxic responses, while Tregs and M2 macrophages promote immunosuppression via TNF-β and IL-10 secretion, collectively enabling immune escape and tumor dissemination.

The microenvironment of lung cancer is a complex ecosystem composed of multiple cell types and extracellular components. It plays a key role in the initiation, progression, metastasis, and treatment response of lung cancer. This microenvironment primarily consists of tumor cells, immune cells, stromal cells, extracellular matrix, and various signaling molecules (**Figure 2**).

### 2.1 Cellular components

The cellular constituents within the lung cancer tumor microenvironment exhibit a high degree of complexity and variability, with the interactions between these cells collectively influencing tumor initiation, progression, and metastatic spread (28). Tumor cells, which serve as pivotal elements within this microenvironment, demonstrate significant capabilities for proliferation, invasion, and extensive metastasis (29). By secreting a range of cytokines and chemokines, including VEGF and platelet-derived growth factor (PDGF), these cells modulate the tumor microenvironment, thereby establishing conditions that favor their own growth and survival (30, 31). Furthermore, tumor cells can undergo an epithelial-mesenchymal transition (EMT), which endows them with mesenchymal traits, ultimately enhancing their migratory and invasive potential, thus facilitating the process of metastasis (32, 33).

Immune cells are integral components of the tumor microenvironment, encompassing a variety of cell types including T cells, B cells, NK cells, macrophages, dendritic cells (DCs), and myeloid-derived suppressor cells (MDSCs) (34). Among these, T cells are pivotal in mediating anti-tumor immunity; specifically, CD8^+^ cytotoxic T lymphocytes are capable of identifying and eliminating tumor cells, thereby exerting significant anti-tumor effects (35). Conversely, regulatory T cells (Tregs) facilitate tumor progression and immune evasion by dampening the activity of diverse immune cell populations and promoting immune tolerance (36). B cells, while known for their role in antibody production and humoral immune responses, exhibit a controversial function within the tumor microenvironment. Some research indicates that B cells can modulate the tumor immune response through cytokine secretion or antigen presentation; however, the nature of these effects—whether they are supportive of tumor growth or detrimental to it—remains a topic of ongoing investigation (37). NK cells are capable of non-specifically targeting and destroying tumor cells, thus playing a crucial role in tumor immune surveillance (38). Nevertheless, the presence of inhibitory factors within the tumor microenvironment, such as transforming growth factor-β (TGF-β) produced by tumor cells, can significantly diminish NK cell functionality and weaken their anti-tumor responses (39, 40).

Macrophages undergo differentiation into tumor-associated macrophages (TAMs) within the tumor microenvironment, which can be categorized into two distinct types: M1 and M2, based on their functional roles and phenotypic characteristics (41). M1-type TAMs exhibit anti-tumor properties and are capable of releasing pro-inflammatory cytokines such as TNF-α and IL-12, which serve to activate immune cells to target and eliminate tumor cells (42). In contrast, M2-type TAMs contribute to tumor progression by secreting immunosuppressive factors, including IL-10 and TGF-β. These factors facilitate tumor cell proliferation, migration, and angiogenesis while concurrently suppressing the host’s anti-tumor immune response (43, 44). Dendritic cells represent the most potent antigen-presenting cells, adept at capturing, processing, and presenting tumor antigens, which is essential for T cell activation and the initiation of anti-tumor immune responses. Nevertheless, various factors present in the tumor microenvironment, such as indoleamine 2,3-dioxygenase (IDO), can hinder the maturation and functional capabilities of DCs, thereby facilitating tumor immune evasion (45, 46). MDSCs comprise a diverse range of cells that significantly accumulate within the tumor microenvironment. These cells suppress immune cell activity through various mechanisms, including the depletion of amino acids, the production of ROS, and the secretion of inhibitory cytokines, all of which contribute to tumor growth and metastatic spread (47, 48).

In the tumor microenvironment, fibroblasts undergo activation and subsequently differentiate into CAFs (49). CAFs modify the tumor microenvironment’s structural integrity by producing extracellular matrix constituents, including collagen and fibronectin, which facilitate the growth and motility of neoplastic cells (50, 51). Furthermore, CAFs release a range of growth factors and cytokines, such as EGF, HGF, and IL-6, thereby enhancing the proliferation, survival, and invasive characteristics of tumor cells. They play a crucial role in modulating the tumor immune microenvironment and fostering angiogenesis within tumors (52, 53). Additionally, CAFs engage in bidirectional interactions with tumor cells, whereby both entities can affect one another’s biological functions through paracrine signaling mechanisms (54).

Vascular endothelial cells play a critical role in the development of blood vessels within tumors. Tumor cells secrete pro-angiogenic factors, including VEGF, which stimulate the proliferation, migration, and formation of lumens in vascular endothelial cells. This process ensures an adequate supply of nutrients and oxygen to tumor tissues, thereby facilitating both tumor growth and metastasis (55). Tumor-associated blood vessels exhibit distinct structural and functional abnormalities, characterized by incomplete vessel walls, heightened permeability, and disorganized blood flow. These imperfections not only compromise blood perfusion in tumors but also create an environment conducive to the dissemination of tumor cells. Additionally, the atypical architecture of these blood vessels plays a role in enabling tumor immune evasion (56, 57). Tumor vascular endothelial cells are capable of expressing various immunomodulatory molecules that further support tumor immune evasion by suppressing the body’s anti-tumor immune response. Within the tumor microenvironment, a complex ecosystem is established through the interactions between vascular endothelial cells and tumor cells (58, 59).

### 2.2 Non cellular components

The tumor microenvironment in lung cancer encompasses various non-cellular elements, including the ECM, cytokines, and chemokines, all of which exert considerable influence on tumor development and the regulation of the microenvironment. The ECM constitutes a sophisticated network comprising components such as collagen, fibronectin, laminin and proteoglycans. This matrix not only offers structural support to tumor cells but is also instrumental in modulating their behavior, including proliferation, migration, invasion, and differentiation (60, 61). Among these components, collagen stands out as a predominant element of the ECM. Alterations in its composition and structural integrity are intricately linked to tumor invasion and metastasis (62, 63). In the context of lung cancer, collagen produced by CAFs can establish a dense fibrous framework that facilitates tumor cell migration. Additionally, it interacts with surface receptors such as integrins on tumor cells, initiating intracellular signaling cascades (for instance, FAK/Src and PI3K/AKT pathways), which in turn enhance tumor cell proliferation, invasion, and the epithelial- EMT (64, 65). Furthermore, glycoproteins such as fibronectin and laminin are crucial for tumor cell adhesion and migration. They facilitate the interaction between tumor cells and the ECM, thereby influencing motility and survival signaling pathways (66, 67). Moreover, proteoglycans, including heparan sulfate proteoglycans, within the ECM can interact with growth factors and cytokines, establishing localized concentration gradients that regulate their activity and distribution. This interaction significantly impacts the signaling networks, angiogenesis, and immune modulation within the tumor microenvironment (68, 69).

Cytokines represent a category of small protein molecules secreted by both immune and tumor cells, playing crucial roles in the regulation of immune responses and cellular growth within the tumor microenvironment of lung cancer (70, 71). Notable cytokines identified in this context include IL, IFN, TNF, and TGF-β. Among these, IL-6 is particularly significant due to its multifunctional nature and high expression levels within the lung cancer tumor microenvironment (72). It facilitates tumor cell proliferation, survival, and migration through the activation of signaling pathways such as signal transducer and activator of transcription 3 (STAT3), concurrently impairing the body’s anti-tumor immune responses (73, 74). Furthermore, IL-6 promotes the differentiation and expansion of Tregs, thereby exacerbating the immunosuppressive conditions present in the tumor microenvironment (75). Interferons, particularly IFN-γ, possess diverse functions including antiviral and anti-tumor capabilities, as well as immune regulation. IFN-γ serves to activate immune cells such as macrophages and NK cells, thereby boosting their anti-tumor efficacy (76, 77). Additionally, it prompts tumor cells to express major histocompatibility complex (MHC) molecules, thereby enhancing tumor immunogenicity and promoting immune surveillance (78). Nevertheless, in a persistent tumor microenvironment, IFN-γ signaling may lead to the expression of immune checkpoint molecules, such as PD-L1, or encourage the infiltration of immunosuppressive cells, establishing a negative feedback loop that hinders immune responses (79). TNF-α functions as a pro-inflammatory cytokine capable of directly inducing tumor cell apoptosis while also exerting indirect anti-tumor effects through the activation of immune cells (80). However, within the tumor microenvironment—especially in lung cancer—TNF-α can stimulate tumor cells to produce anti-apoptotic proteins (e.g., c-FLIP and Bcl-2 family members), promote the release of inflammation-associated factors, drive epithelial- EMT, and bolster the characteristics of tumor stem cells via pathways such as NF-κB. These mechanisms contribute to the survival, invasion, metastasis, and therapeutic resistance of tumor cells (81, 82). TGF-β is a crucial pleiotropic cytokine that typically serves as a potent immunosuppressive and metastasis-facilitating agent in advanced lung cancer (83). TGF-β can directly inhibit the activation and functionality of effector immune cells, including CD8^+^ T cells, NK cells, and macrophages; promote the differentiation, expansion, and functional stability of Treg cells; and induce EMT in tumor cells, thereby enhancing their invasive properties, metastatic potential, stem cell-like characteristics, and resistance to therapy (84–86).

Chemokines represent a specific category of small protein molecules that facilitate the directional movement of immune cells and other cellular types. These proteins are integral to the mobilization of immune cells as well as the metastasis of cancer cells within the tumor microenvironment (87, 88). In the context of lung cancer, the signaling pathway involving chemokines, notably CXC chemokine ligand 12 (CXCL12) and its corresponding receptor, CXC chemokine receptor 4 (CXCR4), is crucial for tumor progression (89). Both tumor cells and tumor-associated fibroblasts have the capacity to secrete CXCL12, which serves to attract tumor cells, immune cells, and vascular endothelial cells that express CXCR4, thereby facilitating their migration toward the tumor site (90, 91). Following the binding of CXCL12 to CXCR4 on the tumor cell surface, the resultant CXCR4-CXCL12 complex activates various intracellular signaling cascades, which in turn enhance the proliferation, migration, and invasion of tumor cells, along with promoting tumor angiogenesis and metastatic spread to distant sites (92, 93). Moreover, the chemokine CCL2 and its receptor CCR2 are critically involved in the recruitment of TAMs (94, 95). CCL2 has the ability to draw CCR2-positive monocytes into the tumor microenvironment, prompting their differentiation into TAMs, ultimately fostering tumor growth and metastatic processes (96, 97) (**Table 1**).

**Table 1:** The main components of the tumor microenvironment of lung cancer.

Main components	Mechanism	Function	References
Cellular components			
T Cell	Identify and kill tumor cells	Anti-tumor effect	(35)
Treg Cell	Inhibit the activity of immune cells and maintain immune tolerance	Promote tumor growth and immune escape	(36)
B Cell	Secreting cytokines or antigen presentation	Affects the immune response of tumors	(37)
M1 macrophages	Secrete pro-inflammatory cytokines, such as TNF - α and IL-12, etc.	Activate immune cells to kill tumor cells	(98)
M2 macrophages	Secrete immunosuppressive factors, such as IL-10 and TGF – β	Promote the proliferation, migration and angiogenesis of tumor cells	(99, 100)
Fibroblast cells	Secrete extracellular matrix components (such as collagen and fibronectin)	Promote the proliferation, survival and invasion of tumor cells and facilitate tumor angiogenesis	(52, 53)
vascular endothelial cell	Induce the proliferation, migration and lumen formation of vascular endothelial cells	Promote the growth and metastasis of tumors	(55)
Non cellular components			
Collagen	Provide scaffolds for the migration of tumor cells	Promote the proliferation, invasion and EMT of tumor cells	(64, 65)
fibronectin	Mediate the interaction between tumor cells and extracellular matrix	Regulate the motility and survival signals of tumor cells	(66, 67)
proteoglycan	Combine growth factors and cytokines to form local concentration gradients	Promote angiogenesis and inhibit immune regulation	(68, 69)
IL-6	Activate Signal Transducer and activator of Transcription 3 (STAT3)	Promote the proliferation, survival and migration of tumor cells, and provide immunosuppression	(73, 74)
IFN-γ	Activate immune cells such as macrophages and NK cells	Enhance anti-tumor activity and promote tumor immune surveillance	(76, 77)
TNF-α	Induce tumor cells to produce anti-apoptotic proteins and promote the release of inflammation-related factors	Support the survival, invasion, metastasis and treatment resistance of tumor cells	(81, 82)
TGF-β	Inhibit the activation and function of effector immune cells such as CD8^+^ T cells, NK cells and macrophages	Enhance the invasion, metastasis, stem cell characteristics and therapeutic resistance of tumor cells	(84–86)
CXCL12	Promote the migration of tumor cells, immune cells, vascular endothelial cells, etc. to the tumor site	Promote the proliferation, migration and invasion of tumor cells	(90, 91)
CXCR4	Promote the proliferation, migration and invasion of tumor cells	Promote the proliferation, migration and invasion of tumor cells	(92, 93)
CCL2	Attract CCR2-positive monocytes into the tumor microenvironment	Promote the growth and metastasis of tumors	(96, 97)

## 3 Mechanisms of epigenetic regulation

**Figure 3:**
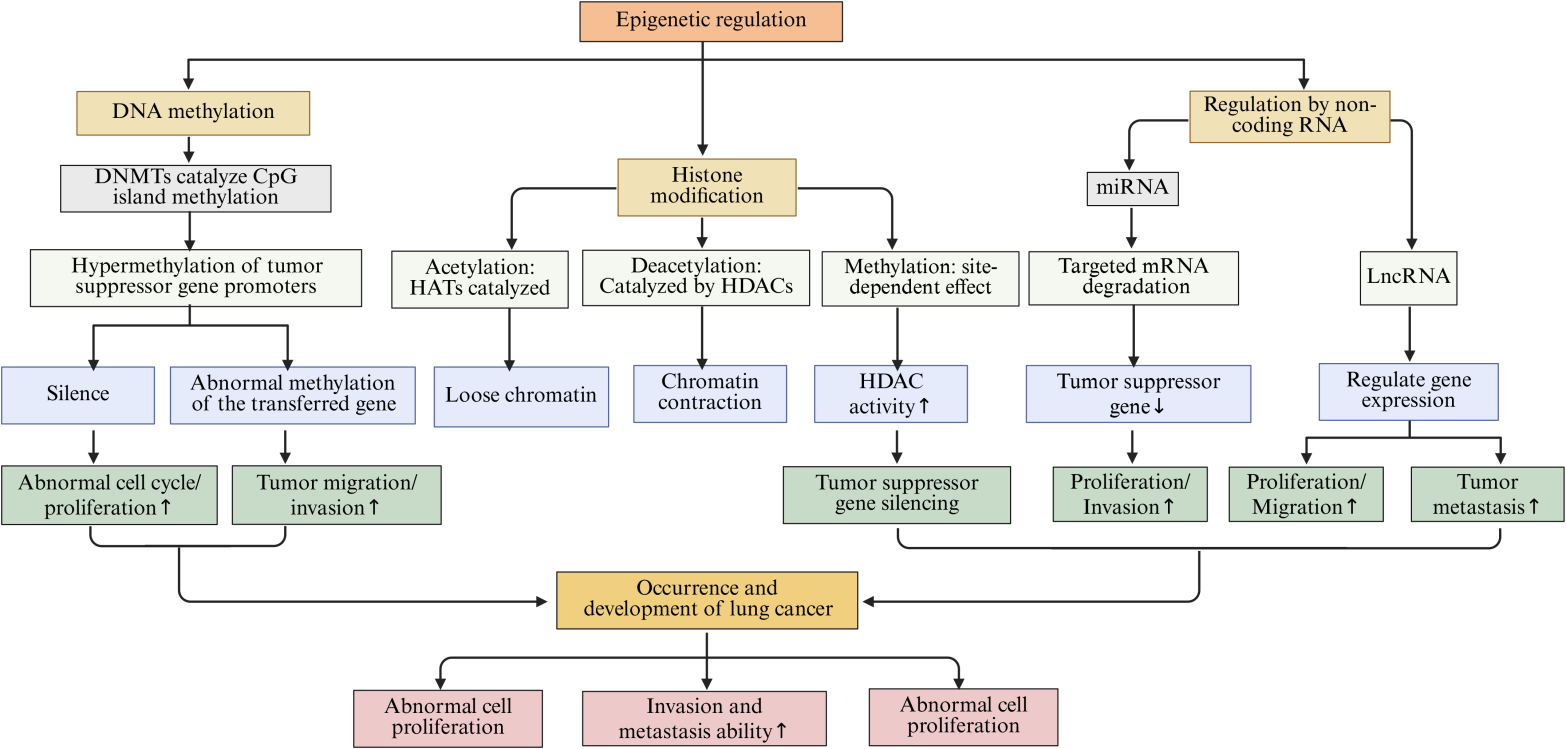
The main epigenetic regulation mechanisms in lung cancer, including DNA methylation, histone modification, and NcRNAs regulation, etc.

Epigenetic regulation pertains to the modulation of gene expression without any modifications to the DNA sequence itself. The primary mechanisms involved in this process encompass DNA methylation, histone modifications, and the regulation by non-coding RNAs (ncRNAs) (101). These mechanisms work in concert to sustain normal cellular operations. However, any disruption in these mechanisms may precipitate the development of various diseases, including lung cancer (**Figure 3**).

DNA methylation refers to the biochemical process whereby methyl groups are added to specific regions of DNA, predominantly within CpG islands, a reaction facilitated by the enzyme DNA methyltransferases (DNMTs) (102). In healthy cells, DNA methylation plays a crucial role in the regulation of gene expression and the maintenance of regular cellular functions (103). Conversely, in the context of lung cancer, the pattern of DNA methylation experiences significant alterations (104). Notably, the promoter regions of numerous tumor suppressor genes are often found in a hypermethylated state, which obstructs the normal transcription and expression of these genes. This disruption results in tumor cells evading their typical growth inhibition mechanisms, thereby acquiring advantages in terms of proliferation and survival (105, 106). Investigations have indicated that the p16 gene frequently undergoes silencing in lung cancer tissues due to hypermethylation of its promoter region, leading to the loss of its regulatory influence on the cell cycle, which in turn fosters the proliferation of lung cancer cells (107, 108). Additionally, DNA methylation has the potential to influence various processes related to metabolism, invasion, and metastasis in tumor cells (109). Studies utilizing lung cancer cell lines have revealed that alterations in the methylation status of specific genes associated with tumor metastasis can significantly impact the migratory and invasive capabilities of tumor cells (110).

Histone modification encompasses the chemical alterations of the amino acid residues within histones, which include processes such as acetylation, methylation, phosphorylation, and ubiquitination (111, 112). These modifications have the capacity to transform the structural and functional properties of chromatin, thereby impacting gene expression (113). Histone acetylation is facilitated by histone acetyltransferases (HATs), which relax the chromatin architecture, enhance the accessibility of genes, and stimulate transcriptional activity (114). Conversely, histone deacetylation is mediated by histone deacetylases (HDACs), resulting in a more compact chromatin structure that suppresses gene transcription (115). In the context of lung cancer, the equilibrium between histone acetylation and deacetylation is perturbed, resulting in the dysregulation of gene expression (116, 117). Research has indicated that the heightened activity of HDACs in lung cancer cells correlates with increased levels of histone deacetylation, leading to the repression of certain tumor suppressor genes and consequently facilitating the onset and progression of lung cancer (118). The modification of histone methylation is characterized by its complexity, as varying locations and extents of methylation can elicit diverse biological outcomes that may either promote or inhibit gene expression. Moreover, its specific implications in lung cancer are contingent upon the location and extent of modification, along with the associated regulatory elements (119, 120).

Non-coding RNAs (ncRNAs) represent a category of RNA molecules that lack protein-coding capabilities, encompassing microRNAs (miRNAs) and long non-coding RNAs (lncRNAs). These molecules are integral to epigenetic regulation (121). miRNAs are characterized as small RNA entities, approximately 22 nucleotides in length, that modulate gene expression through complementary binding to the mRNA of specific target genes. This interaction can suppress the translation of mRNA or facilitate its degradation (122, 123). In the context of lung cancer, numerous miRNAs exhibit aberrant expression patterns that are implicated in various cellular processes, including proliferation, apoptosis, invasion, and metastasis of lung cancer cells (124). Notably, miR-21 is found to be overexpressed in lung cancer tissues, where it can target and downregulate multiple tumor suppressor genes, thereby enhancing the proliferation and invasiveness of lung cancer cells (125). lncRNA is a ncRNAs with a length of more than 200 nucleotides. lncRNA’s mechanism of action is more complex. It can regulate gene expression at multiple levels, including the transcriptional and post-transcriptional levels, by interacting with DNA, RNA, or proteins (126, 127). Within lung cancer pathology, certain lncRNAs, such as MALAT1 and HOTAIR, are significantly associated with the onset and progression of the disease (128). MALAT1 has been shown to facilitate the proliferation, migration, and invasion of lung cancer cells by modulating the expression of associated genes (129). Conversely, HOTAIR interacts with chromatin modification complexes to modify chromatin states, which subsequently affects gene expression and promotes metastasis in lung cancer (130).

## 4 Epigenetic regulatory mechanisms in the microenvironment of lung cancer

### 4.1 The regulatory role of DNA methylation in the microenvironment of lung cancer

DNA methylation is an epigenetic modification catalyzed by DNA methyltransferases (DNMTs). This modification regulates gene expression by adding methyl groups to CpG islands and serves as an important mechanism for maintaining genomic stability and influencing cell differentiation and survival (103). Specifically, in the lung cancer microenvironment, abnormal changes in DNA methylation have a profound impact on gene expression and cell function. These alterations play a key role in the occurrence, development, metastasis, and response to treatment of lung cancer.

In the process of lung cancer development, abnormal DNA methylation is manifested not only as hypermethylation of individual tumor suppressor gene promoters but also as epigenetic reprogramming driven by a systemic imbalance in the expression or activity of enzymes regulating DNA methylation (131). DNA methyltransferases (DNMTs) are the main writers, including DNMT1, DNMT3A, and DNMT3B, which achieve gene silencing by catalyzing the transfer of a methyl group to cytosine, resulting in the formation of 5-methylcytosine (5mC) (132). Studies have shown that overexpression of DNMT1 leads to methylation-mediated silencing of promoter regions of multiple tumor suppressor genes (such as p16, RASSF1A, and CDH1), thereby inhibiting apoptosis and promoting tumor cell proliferation and invasion (133). The p16 tumor suppressor gene is pivotal in the regulation of the cell cycle. The protein encoded by this gene serves to inhibit the activity of cyclin-dependent kinases 4 and 6 (CDK4/6), thereby obstructing the transition of cells from the G1 phase to the S phase and consequently suppressing cellular proliferation (134). Within lung cancer cells, the promoter region of the p16 gene frequently experiences hypermethylation, which leads to transcriptional silencing of the gene. As a result, the inhibition of CDK4/6 is lost, causing the cell cycle to become dysregulated. This allows lung cancer cells to continuously proliferate, facilitating tumor initiation and progression (135). Research indicates that in cases of non-small cell lung cancer, the prevalence of hypermethylation at the p16 gene promoter can range from 50% to 70%, correlating closely with the tumor’s stage, grade, and patient prognosis (104, 136). Another frequently silenced tumor suppressor gene, RASSF1A, also undergoes promoter hypermethylation. The protein produced by RASSF1A is integral to various cellular processes, including apoptosis, regulation of the cell cycle, and the inhibition of tumor cell migration and invasion (137). In lung cancer, hypermethylation of the RASSF1A promoter results in the loss of its expression, allowing cancer cells to evade apoptosis while enhancing their proliferative and metastatic potential (138). A comprehensive study involving a large cohort of lung cancer patients revealed that those exhibiting hypermethylation of the RASSF1A promoter had significantly poorer 5-year survival rates compared to their counterparts without such hypermethylation (139). In addition, epigenetic silencing of the CDH1 gene by methylation is of significant importance in lung cancer. The adhesion molecule E-cadherin, encoded by CDH1, maintains intercellular connections and tissue structural integrity of epithelial cells. Methylation of its promoter can lead to loss of protein expression, thereby weakening intercellular adhesion and promoting EMT (140). This process not only enhances the migration and invasion capabilities of lung cancer cells but also promotes tumor immune evasion by regulating immune-related signals. For example, loss of E-cadherin can upregulate PD-L1 expression and reshape the tumor immune microenvironment, enabling tumor cells to evade immune surveillance (141). Therefore, CDH1 methylation is not only a hallmark event of tumor invasion and metastasis but also reflects the functional cross-talk between epigenetic regulation and immune evasion. Furthermore, abnormal activation of DNMT3A and DNMT3B is closely related to the early occurrence of lung cancer, and their inhibitors (such as azacitidine and decitabine) have been approved for use in hematological tumors and are being explored for epigenetic therapy in lung cancer (142). In contrast, members of the demethylase (erasers) family, such as TET1, TET2, and TET3, promote the demethylation process by oxidizing 5-methylcytosine (5mC) to 5-hydroxymethylcytosine (5hmC). Loss of their function often leads to global reduction of 5hmC and hypermethylation at specific loci (143). For example, TET2 mutations or downregulation can promote the maintenance of cancer stemness and tumor immune evasion in lung cancer cells. Additionally, loss of TET1 enhances tumor adaptive metabolism by upregulating hypoxia-inducible factor 1-alpha (HIF-1α) signaling (144).

Beyond its effects on tumor cells directly, DNA methylation is also integral to intercellular signaling and immune modulation within the tumor microenvironment of lung cancer (145). TAMs represent a significant subset of immune cells present in this environment, and their functional characteristics are pivotal for tumor progression (146). Studies have demonstrated that the DNA methylation patterns of specific genes within TAMs can influence their polarization and operational capacities. For example, the hypomethylation observed in the promoter region of the Arg1 gene leads to an upregulation of this gene’s expression, thereby steering TAMs toward an M2 polarization. M2-type TAMs are characterized by their immunosuppressive roles and their ability to secrete a variety of cytokines, including IL-10 and TGF-β, which can inhibit the functions of immune cells such as T cells and NK cells, thus promoting tumor immune evasion (147). In contrast, the restoration of TET enzyme activity can enhance T cell infiltration by remodeling the chemokine network, thereby improving the response rate to immune checkpoint therapy (148). Conversely, DNMT1-mediated methylation silences chemokines such as CXCL9 and CXCL10, leading to reduced anti-tumor immune cell infiltration; the restoration of TET1 and TET2 can reverse this effect and improve the efficacy of immune checkpoint inhibitors (ICIs) (149). In addition, abnormal DNA methylation in cancer-associated fibroblasts (CAFs) promotes extracellular matrix stiffening and tumor angiogenesis by upregulating extracellular matrix remodeling-related genes, such as COL1A1 and LOXL2, and increasing growth factor secretion. This creates a more invasive niche for cancer cells (150, 151). Beyond TAMs and CAFs, remodeling of DNA methylation in other immune cells also contributes to reprogramming the immune microenvironment in lung cancer (152). In T cells, epigenetic remodeling induced by chronic antigen stimulation fixes the exhausted phenotype. DNMT3A-mediated methylation of the promoters of PDCD1 (PD-1), LAG3, and HAVCR2 (TIM-3) leads to sustained high expression of inhibitory receptors, resulting in functional exhaustion of CD8^+^T cells; in contrast, activation of TET2 and TET3 can restore the memory phenotype and enhance the response to immune checkpoint therapy (153). In dendritic cells (DCs), overactivation of DNMT1 and DNMT3B causes hypermethylation of genes such as MHC-II, CD80/CD86, and IFN-β, inhibiting antigen presentation and T cell activation. Restoration of TET2 can reconstruct the inflammatory transcription program and enhance anti-tumor immunity (154). In tumor-associated neutrophils (TANs), abnormal methylation of IL-8, CXCR2, and PAD4 mediated by DNMT1 and DNMT3A drives N2 polarization, promoting angiogenesis and immune suppression; activation of TET3 can revert this phenotype to N1, enhancing reactive oxygen species-dependent tumor-killing effects (155).

During the metastasis of lung cancer, DNA methylation also plays an important role. EMT is a key process for tumor cells to acquire invasive and metastatic capabilities (156). Studies have shown that DNA methylation can influence the metastatic ability of lung cancer cells by regulating the expression levels of EMT-related genes (157). Transcription factors such as Snail and Slug are key regulatory factors in the EMT process. During the metastasis of lung cancer cells, the methylation status of the promoter regions of their genes undergoes hypomethylation, which leads to the upregulation of these transcription factors (158). Low methylation of the Snail gene promoter increases its expression. The Snail protein can bind to the promoter region of the E-cadherin gene and inhibit its expression, which decreases cell-cell adhesion among lung cancer cells. Additionally, Snail promotes the expression of mesenchymal markers such as N-cadherin and vimentin. This allows lung cancer cells to acquire mesenchymal characteristics, making them more prone to invasion and metastasis (159). Furthermore, studies have found that the methylation state of certain miRNA genes related to tumor metastasis is closely associated with lung cancer metastasis (160). The miR-34a gene is often downregulated in lung cancer due to high methylation of its promoter. miR-34a targets and inhibits several genes associated with tumor metastasis. Following miR-34a downregulation, its inhibitory effect on target genes weakens, leading to enhanced invasion and metastatic ability of lung cancer cells (161–163).

**Figure 4:**
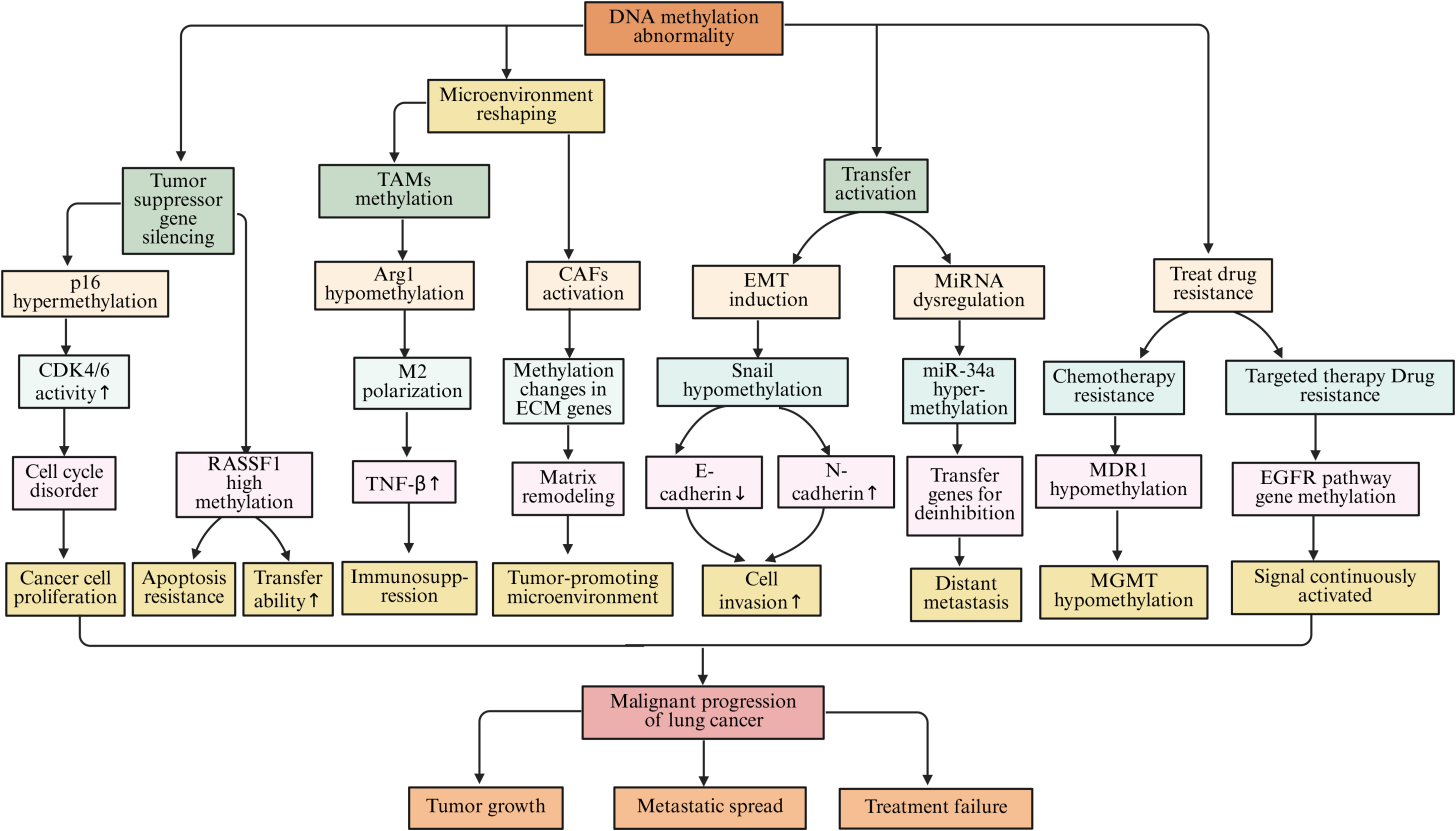
The regulatory role of DNA methylation in the microenvironment of lung cancer.

DNA methylation is closely related to the treatment resistance of lung cancer. During chemotherapy for lung cancer, tumor cells can alter the expression of resistance-related genes through DNA methylation, leading to resistance (164, 165). Multidrug resistance gene 1 (MDR1) encodes P-glycoprotein (P-gp), a drug efflux pump that can expel chemotherapy drugs that enter the cell, resulting in tumor cells developing resistance to chemotherapy drugs (166). Studies have found that after long-term stimulation with chemotherapy drugs, the methylation level of the MDR1 gene promoter region decreases, leading to upregulation of gene expression and overexpression of P-gp protein, causing lung cancer cells to develop resistance to various chemotherapy drugs, such as paclitaxel and cisplatin (167, 168). O6-methylguanine-DNA methyltransferase (MGMT) is a DNA repair enzyme, and its promoter methylation status is closely related to the sensitivity of tumor cells to alkylating agent chemotherapy drugs (169). When the MGMT promoter is highly methylated, gene expression is suppressed, and tumor cells become more sensitive to alkylating agents such as temozolomide. Conversely, when the MGMT promoter is lowly methylated, gene expression is upregulated, allowing tumor cells to repair DNA damage caused by alkylating agents, leading to resistance to these drugs (170, 171). In targeted therapy for lung cancer, DNA methylation can also affect treatment outcomes. For example, during treatment of non-small cell lung cancer with epidermal growth factor receptor (EGFR) tyrosine kinase inhibitors (TKI), some patients may develop resistance (172). Studies have found that the methylation status of some genes related to the EGFR signaling pathway changes in resistant cells, leading to sustained activation of the EGFR signaling pathway, causing tumor cells to develop resistance to TKI (173, 174) (**Figure 4**).

### 4.2 The regulatory role of histone modifications in the microenvironment of lung cancer

**Figure 5:**
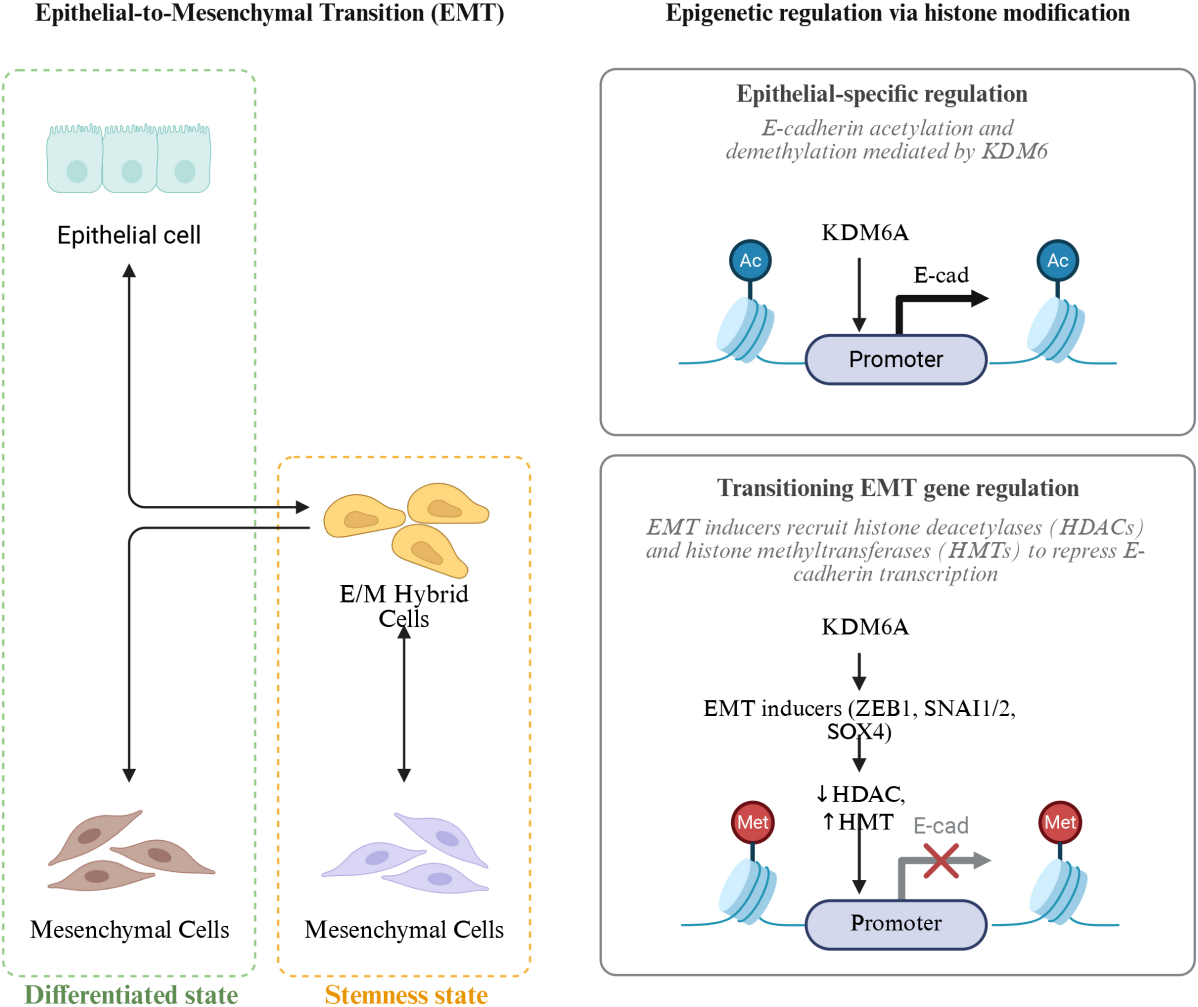
Epigenetic regulation of epithelial-to-mesenchymal transition (EMT) via histone modification. EMT is a dynamic process in which epithelial cells acquire mesenchymal features through intermediate E/M hybrid states, balancing differentiation and stemness. Histone modifications play a key role in this transition: KDM6A mediates E-cadherin (E-cad) acetylation and demethylation to maintain epithelial identity, whereas EMT inducers (ZEB1, SNAI1/2, SOX4) recruit histone deacetylases (HDACs) and histone methyltransferases (HMTs) to repress E-cad transcription, promoting mesenchymal conversion and enhanced cellular plasticity.AC (Acetylation).

Histone modifications, as an important mechanism of regulation at the chromatin level, play a key role in the remodeling of chromatin structure and the activation or silencing of gene transcription in the lung cancer microenvironment. Chromatin is formed by DNA wrapping around histone octamers to create nucleosomes, which are further assembled into higher-order structures (175). Within these nucleosomes, the N-terminal tails of histones extend out from the surface, and the amino acid residues on them can undergo various post-translational modifications, including acetylation, methylation, phosphorylation, and ubiquitination. These modifications can alter the compactness and accessibility of chromatin, thereby affecting gene transcription activity (176). Although histone modifications are post-translational modifications of proteins at the molecular level, they are often regarded as core components of epigenetic mechanisms. This is because these modifications can regulate gene expression in a heritable and reversible manner without changing the DNA sequence, and they have long-term regulatory effects in cell differentiation, stress response, and tumor microenvironment remodeling (177) (**Figure 5**).

Histone acetylation and deacetylation represent dynamic and regulated modifications that are orchestrated by HATs and histone deacetylases (HDACs), respectively (178). In healthy cellular contexts, this equilibrium plays a crucial role in sustaining appropriate gene expression. However, within the lung cancer microenvironment, this delicate balance is frequently disturbed. Research indicates that lung cancer cells often exhibit elevated levels of both the expression and activity of HDACs. These enzymes facilitate the removal of acetyl groups from histones, which subsequently results in chromatin condensation and the repression of gene transcription (179, 180). For instance, the upregulation of HDACs leads to histone deacetylation at the promoter regions of critical tumor suppressor genes, including p53 and Rb, thereby contributing to a tighter chromatin configuration. As a result, the transcriptional activity of these tumor suppressor genes is inhibited, undermining their essential roles in curbing tumor cell proliferation and fostering apoptosis, which ultimately promotes the progression of lung cancer (181). Conversely, a reduction in HAT activity or expression leads to diminished levels of histone acetylation, further disrupting normal gene expression (182). In light of these observations, various studies have investigated the therapeutic efficacy of histone deacetylase inhibitors (HDACi) in the treatment of lung cancer. These inhibitors function by obstructing HDAC activity, enhancing histone acetylation, and reactivating silenced tumor suppressor genes, consequently thwarting the growth and proliferation of lung cancer cells (183). In both lung cancer cell lines and animal models, HDACi have been shown to induce apoptosis, halt cell cycle progression, and partially inhibit tumor growth and metastasis (184).

Histone methylation modifications are relatively complex and can occur on different amino acid residues of histones, with varying degrees of methylation (monomethylation, dimethylation, and trimethylation). The different modification sites and degrees have varying effects on gene transcription (185). In the lung cancer microenvironment, abnormal changes in histone methylation contribute significantly to the occurrence and progression of lung cancer (186). For example, trimethylation at lysine 4 of histone H3 (H3K4me3) is usually associated with gene activation. In lung cancer, certain genes that promote tumor growth and metastasis have increased H3K4me3 marks in their promoter regions, enhancing the transcriptional activity of these genes (187). Studies have found that in NSCLC, some angiogenesis-related genes, such as the VEGF gene, exhibit elevated H3K4me3 modification in their promoter regions, promoting VEGF gene transcription. This facilitates tumor angiogenesis, providing sufficient nutrients and oxygen for tumor cells, thereby supporting tumor growth and metastasis (188). Conversely, trimethylation at lysine 27 of histone H3 (H3K27me3) is usually associated with gene silencing (189). Moreover, in lung cancer, some tumor suppressor genes show enhanced H3K27me3 levels in their promoter regions. This leads to the silencing of these genes. For instance, the increased H3K27me3 modification in the promoter region of the p16 gene prevents its normal expression. This results in the loss of its regulatory effect on the cell cycle, giving lung cancer cells a proliferative advantage (190). Histone methylation is catalyzed by histone methyltransferases (HMTs), with different HMTs responsible for methylation modifications at various sites and degrees (191). In lung cancer, abnormal expression of multiple HMTs leads to disrupted methylation patterns, among which EZH2 and SETD2 are the most representative key regulators. EZH2, the core methyltransferase of the polycomb repressive complex PRC2, is capable of catalyzing the H3K27me3 modification (192). EZH2 is commonly upregulated in lung cancer. Excessive H3K27me3 silences tumor suppressor genes including p16, CDH1, and PTEN, promoting cell proliferation, EMT, and invasive metastasis (193). Additionally, EZH2 has multiple immunoregulatory effects in the TME. On one hand, EZH2 promotes immune evasion by silencing the expression of MHC-I and T cell chemokines (such as CXCL9 and CXCL10), reducing antigen presentation and CD8^+^T cell infiltration (194). On the other hand, EZH2 can maintain the immunosuppressive phenotype of Treg cells by silencing cytotoxic factor gene expression via methylation, such as IFNG and GZMB, weakening the effector function of CD8^+^T cells (195, 196). Meanwhile, in DCs, overactivation of EZH2 can inhibit the expression of co-stimulatory molecules (CD80/CD86) and antigen presentation molecules, limiting the activation of naïve T cells. Its inhibition can restore DC-mediated immunogenic responses (197). In TANs, EZH2-mediated H3K27me3 enrichment is associated with the silencing of pro-inflammatory factors (such as IL-12 and TNF-α), promoting N2 polarization and angiogenesis. EZH2 inhibitors can reverse this phenotype, enhancing N1 cytotoxic function and immune response (198). Furthermore, EZH2 collaborates with histone demethylase LSD1 to co-regulate the chromatin accessibility of T cell exhaustion markers. An imbalance in their activities can lead to stabilization of T cell exhaustion and reduced response to anti-PD-1 therapy (199). In mouse lung cancer models, EZH2 inhibitors (such as Tazemetostat) can significantly reduce H3K27me3 levels, relieve the epigenetic silencing of antigen presentation genes, and synergize with PD-1/PD-L1 immune checkpoint inhibitors, suggesting the potential of combining epigenetic therapy with immunotherapy (200). In contrast, SETD2 is the only H3K36me3 methyltransferase in mammals, and its function is crucial for maintaining genomic stability, RNA splicing accuracy, and DNA repair (201). In lung cancer, mutations or deletions of SETD2 lead to decreased H3K36me3 levels, triggering replication stress, DNA mismatch repair defects, and genomic instability, thereby promoting tumor heterogeneity and drug resistance (202). More importantly, downregulation of SETD2 weakens the transcriptional activation of immune-related genes and reduces neoantigen presentation and interferon signaling. It also inhibits CD8^+^T cell infiltration and enhances the accumulation of immunosuppressive cells, such as Tregs and M2 TAMs (203). However, impaired SETD2 function can activate the cGAS-STING pathway and type I interferon signaling, triggering a “double-edged sword” effect characterized by both chronic inflammation and immune exhaustion. Clinically, SETD2 mutations are considered associated with unstable efficacy of immune checkpoint inhibitors and are potential targets for immune profiling combined with epigenetic therapy (204, 205).

**Figure 6:**
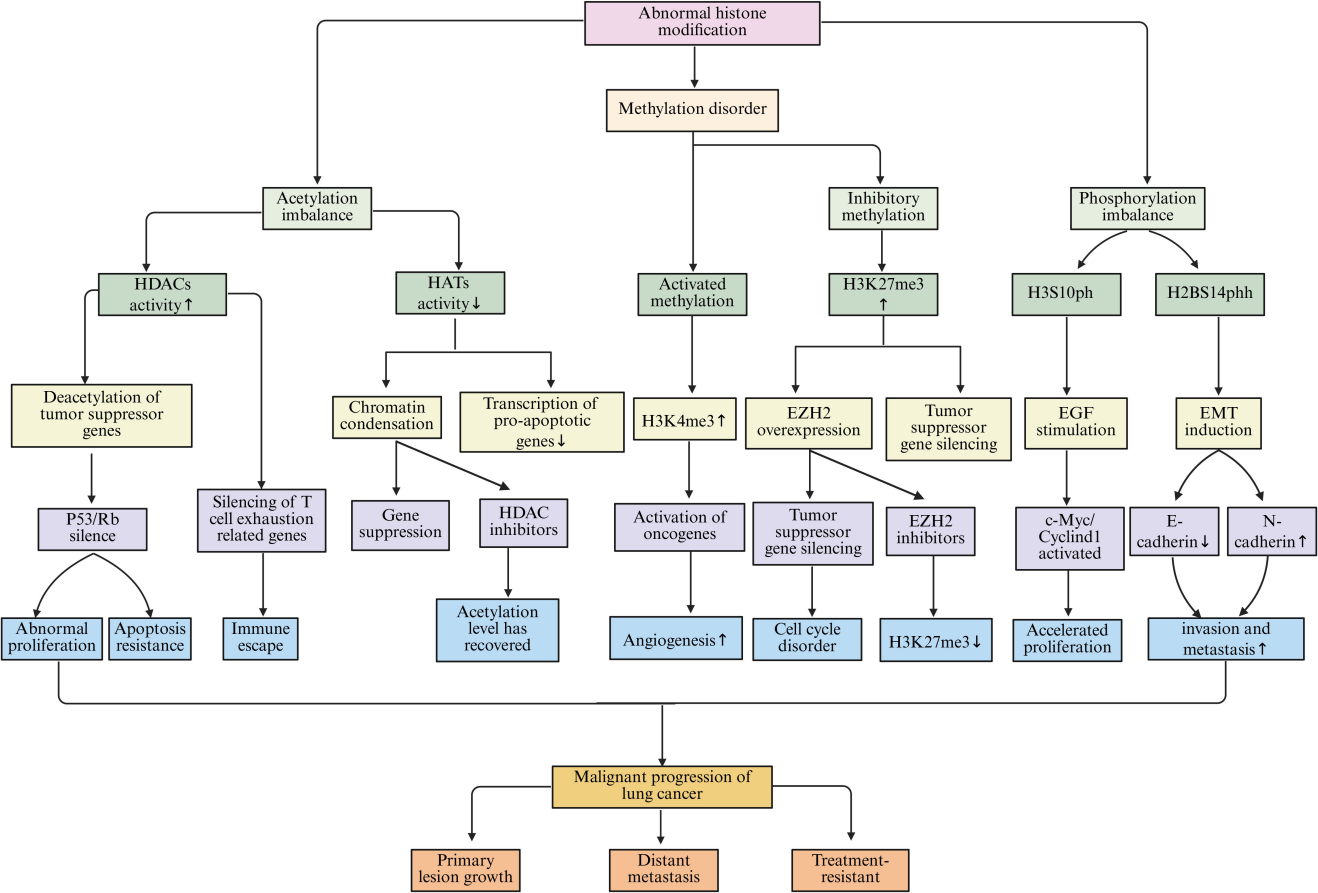
The regulatory role of histone modification in the microenvironment of lung cancer.

Histone phosphorylation is the addition of phosphate groups to specific amino acid residues of histones under the action of protein kinase. This modification can alter the interaction between histones and DNA, affecting the structure and function of chromatin, thereby regulating gene transcription (206). Such phosphorylation events play important roles in the lung cancer microenvironment, being involved in the initiation and progression of lung cancer. Studies have found that lung cancer cells, when stimulated by external factors such as growth factors and cytokines, exhibit phosphorylation of histones H2A, H2B, H3, and H4 (207). Among these, phosphorylation at serine 10 of histone H3 (H3S10ph) is closely associated with gene activation. Specifically, in lung cancer cells stimulated by EGF, intracellular signaling pathways are activated, leading to increased protein kinase activities and resulting in H3S10 phosphorylation (208). H3S10ph can alter the structure of chromatin and promote the transcription of genes related to cell proliferation and survival, such as c-Myc and Cyclin D1, thereby facilitating the proliferation and survival of lung cancer cells (209, 210). Histone phosphorylation is also linked to the invasion and metastasis capabilities of lung cancer cells. During the EMT process in lung cancer cells, specific histone phosphorylation modifications change (211). Research shows that under the stimulation of EMT-inducing factors, such as TGF-β, the phosphorylation level of serine 14 on histone H2B (H2BS14ph) increases (212). H2BS14ph can regulate the expression of EMT-related genes by affecting the recruitment of chromatin remodeling complexes, promoting the expression of mesenchymal markers like N-cadherin and vimentin, while inhibiting the expression of epithelial markers like E-cadherin. This allows lung cancer cells to acquire mesenchymal characteristics and enhances their invasion and metastasis capabilities (213) (**Figure 6**).

### 4.3 Regulatory role of Non-coding RNA in lung cancer microenvironment

Non-coding RNA refers to a category of RNA molecules that do not translate into proteins, yet they serve an essential regulatory function within the microenvironment of lung cancer. This category predominantly encompasses miRNA and lncRNA. These molecular entities modulate gene expression at both transcriptional and post-transcriptional stages through their interactions with specific target genes. Consequently, they impact the biological activities of lung cancer cells as well as the cellular communication and interactions occurring within the lung cancer microenvironment.

miRNAs are small RNA molecules approximately 22 nucleotides in length. They bind to the 3’-untranslated region (3’-UTR) of target gene mRNA through complementary pairing, inhibiting mRNA translation or promoting its degradation, thereby achieving negative regulation of gene expression (214). In the lung cancer microenvironment, the expression levels of many miRNAs are abnormally altered. These miRNAs participate in the processes of lung cancer occurrence, development, metastasis, and immune escape (215). miR-21 is highly expressed in lung cancer tissues and cell lines. It can target and inhibit the expression of tumor suppressor genes such as programmed cell death protein 4 (PDCD4), and phosphatase and tensin homolog (PTEN) (216). After the expression of PDCD4 is suppressed, PDCD4 cannot effectively inhibit the activity of the protein translation initiation factor eIF4A, which promotes the proliferation and invasion of lung cancer cells (217). The downregulation of PTEN expression weakens its inhibitory effect on the phosphoinositide 3-kinase (PI3K)/protein kinase B (AKT) signaling pathway. This activates the signaling pathway and enables lung cancer cells to acquire stronger survival and proliferation capabilities (218). miR-155 is also highly expressed in the lung cancer microenvironment. It can affect the function of immune cells by targeting and regulating some immune-related genes (219). miR-155 can target and inhibit the expression of the SHIP1 gene, which is a negative regulator involved in immune cell signaling. Its suppression leads to excessive activation of immune cells and enhanced inflammatory responses, facilitating immune escape of lung cancer cells (220, 221). miR-155 can also promote the polarization of TAMs towards the M2 type, enhancing the immunosuppressive function of TAMs and further inhibiting the body’s anti-tumor immune response (222). In addition to TAMs, miRNAs also mediate epigenetic regulation and immune exhaustion in various immune populations. For example, in T cells, miR-31, miR-146a, and miR-155 can target molecules such as T-bet, SOCS1, and DNMT1, reshaping exhaustion-related epigenetic programs (223). Upregulation of miR-31 promotes the expression of TOX and PD-1, locking CD8^+^T cells into an exhausted phenotype. Upregulation of miR-146a inhibits IFN-γ production, weakening cytotoxic responses, while miR-34a targets PD-L1 mRNA, partially restoring the killing activity of T cells and enhancing sensitivity to immune checkpoint therapy (224, 225). In DCs, the miR-148/152 family enhances antigen presentation by inhibiting CaMKIIα and DNMT1, upregulating CD80/CD86 and type I interferons; conversely, high expression of miR-22 and miR-146a inhibits IRF5, RelB, and IL-12, inducing DCs to transition to a tolerant phenotype, weakening T cell activation (226). In TANs, miR-223 and miR-142-3p regulate the STAT3 and HIF-1α pathways, balancing pro-inflammatory and immunosuppressive responses. Downregulation of miR-223 drives N2 polarization and enhances VEGF expression, promoting angiogenesis and immune evasion, while upregulation of miR-142-3p can inhibit Neutrophil Extracellular Traps (NETs) formation and restore pro-inflammatory responses (227, 228). Furthermore, an increasing number of studies have found that miRNAs can directly regulate epigenetic mechanisms. They become key nodes connecting metabolism and immune remodeling. For example, the miR-29 family can target DNA methyltransferases DNMT3A/3B, downregulating their expression, thereby relieving the methylation silencing of tumor suppressor genes, such as p15 and RASSF1A. miR-148a and miR-152 directly target DNMT1, restoring gene demethylation and inhibiting the EMT process in lung cancer cells. miR-101 and miR-26a can downregulate histone methyltransferase EZH2, reducing H3K27me3 levels and reactivating the transcription of tumor suppressor genes, thereby enhancing T cell activation and anti-tumor immune responses (229, 230). Additionally, miR-34a targets deacetylase SIRT1, increasing p53 acetylation levels, promoting cell cycle arrest and apoptosis (231). miR-137 inhibits histone demethylase LSD1 (KDM1A), blocking the EMT process in lung cancer cells (232). These miRNAs reshape chromatin states and the immune microenvironment through the “miRNA–epigenetic enzyme” axis, revealing the molecular intersection of immune evasion and metabolic abnormalities in lung cancer.

LncRNAs are classified as non-coding RNAs that exceed 200 nucleotides in length. The mechanisms by which they exert their effects are intricate, allowing them to modulate gene expression through a variety of pathways (233). Within the lung cancer microenvironment, lncRNAs engage with DNA, RNA, and proteins, fulfilling regulatory functions at several tiers, encompassing both transcriptional and post-transcriptional levels (234). One notable lncRNA, MALAT1, has been closely associated with the metastasis of lung cancer and exhibits elevated expression levels in lung cancer tissues and cellular models (235). MALAT1 regulates the expression of genes associated with tumor metastasis by recruiting chromatin modification complexes and altering the structure and accessibility of chromatin (236). MALAT1 can interact with the histone methyltransferase EZH2, leading to the enrichment of EZH2 in certain tumor suppressor gene promoter regions and catalyzing the trimethylation of H3K27me3. This results in the silencing of these tumor suppressor genes and promotes the invasion and metastasis of lung cancer cells. Moreover, MALAT1 affects the stability and transport of specific mRNAs related to cell migration and invasion, enhancing their stability and thereby regulating the biological behavior of lung cancer cells (237, 238). Similarly, HOTAIR is also a LncRNA that plays an important role in lung cancer; it regulates gene expression by interacting with various proteins and RNAs (239). HOTAIR binds to the PRC2 complex, recruiting PRC2 to specific gene loci and promoting gene silencing (240). In lung cancer, HOTAIR promotes tumor occurrence and development by regulating the expression of genes related to cell proliferation, apoptosis, and metastasis (241). Research shows that HOTAIR can inhibit the expression of the E-cadherin gene, promoting the EMT process of lung cancer cells and enabling them to acquire stronger invasion and metastasis capabilities (238). Further research indicates that some lncRNAs directly affect the stability or recruitment of epigenetic enzymes, thereby altering the chromatin accessibility in lung cancer cells and immune cells. For example, the XIST–EZH2 axis involves XIST recruiting EZH2 to mediate H3K27me3-dependent silencing of CDH1 and KLF2, which promotes the proliferation and migration of lung cancer cells (242). The NEAT1-EZH2/DNMT1 axis functions through NEAT1 binding to EZH2 or DNMT1, leading to silencing of p21 and DUSP4 by H3K27me3 or DNA methylation, facilitating tumor immune evasion and chemotherapy resistance (243). In the PVT1-EZH2 axis, PVT1 stabilizes EZH2 protein levels and suppresses the expression of the miR-200 family of microRNAs and tumor suppressor genes, inducing an immunosuppressive phenotype (244). The LINC01138-PRMT5 axis involves LINC01138 binding to and stabilizing the arginine methyltransferase PRMT5, thereby potentiating gene silencing and immunosuppressive transcriptional programs (245). Lastly, the SNHG16–HDAC1 axis recruits HDAC1 to deacetylate the p53 promoter, reducing its transcriptional activity and promoting cell survival and immune tolerance (246).

**Figure 7:**
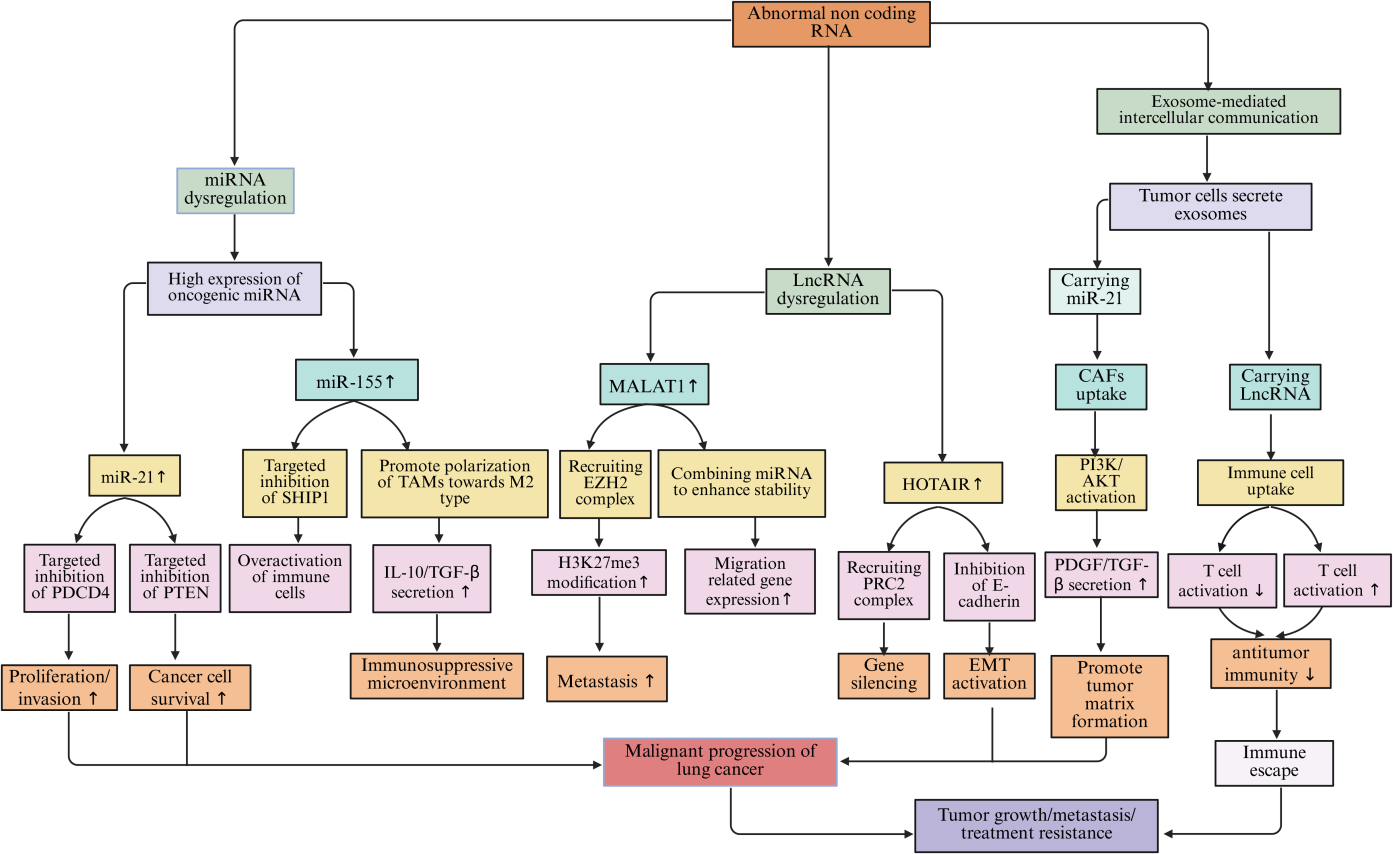
Regulatory role of ncRNAs in the microenvironment of lung cancer.

Non-coding RNAs play a key role in intercellular communication within the lung cancer microenvironment. Tumor cells can transfer their ncRNAs to surrounding cells through the secretion of exosomes, thereby affecting their functions (247). Lung cancer exosomes contain a large amount of miRNAs and lncRNAs, which can be taken up by CAFs and immune cells (248). Exosomal miR-21 can be taken up by CAFs, activating the PI3K/Akt pathway, leading CAFs to secrete more extracellular matrix and pro-tumor factors (such as TGF-β, PDGF), providing a more favorable growth environment for tumor cells (249). Meanwhile, ncRNAs carried by exosomes, such as miR-29, miR-101, MALAT1, and HOTAIR, can directly regulate epigenetic modifications in recipient cells. For example, in T cells, exosomal miR-214 and miR-24-3p inhibit signaling transduction and methylation regulation by targeting PTEN/DNMT1, inducing the expression of exhaustion genes (PDCD1, TOX); while miR-34a and miR-101 downregulate EZH2 and reduce H3K27me3, partially restoring cytotoxic activity (250). In DCs, exosomal HOTAIR recruits DNMT1 to inhibit CD80/CD86 and IL-12, inducing immune tolerance; while miR-148a/152 inhibits DNMT1 to promote antigen presentation and type I interferon secretion (251). In TANs, exosomal miR-223 and LINC00665 activate the STAT3/NF-κB pathway, driving N2 polarization and enhancing VEGF and IL-10 expression; conversely, miR-142-3p downregulates PAD4 to inhibit NETs formation, improving the pro-inflammatory immune environment (252) (**Table 2**, **Figure 7**).

**Table 2:** Regulatory network of ncRNA-mediated epigenetic and immune modulation in lung cancer TME.

Type	Represents ncRNA	Main target/pathway	Epigenetic mechanism	Target cell	Function and immune effects
miRNA	miR-21	PTEN, PDCD4	Inhibit the PI3K/Akt inhibitory factor →sustained signaling activation	Lung cancer cells/CAFs	Promote tumor growth and invasion; CAFs secrete ECM, TGF-β, PDGF
	miR-155	SHIP1, SOCS1	Regulating negative feedback of immune signals and affecting polarization	TAMs/T cells	Promote M2 polarization and immune suppression; Inducing T cell exhaustion
	miR-31, miR-146a	T-β, IFNG	Alter the methylation of genes related to T cell exhaustion	T cells	Promote the expression of PD-1 and TOX, and weaken cytotoxic responses
	miR-34a	PD-L1, SIRT1	Deacetylation regulation Enhance p53 activity	Tumor cells/T cells	Restore anti-tumor immunity and cell cycle arrest
	miR-29 family	DNMT3A/3B	Remove the methylation silencing of tumor suppressor genes	Lung cancer cells	Inhibit proliferation and promote the recovery of gene expression
	miR-101/miR-26a	EZH2	Reduce H3K27me3 level	Tumour cells/T cells	Restart tumor suppressor genes to enhance immune activation
	miR-148a/miR-152	DNMT1	Demethylated antigen-presenting genes	DCs	Enhance type I interferon and T cell activation
	miR-223/miR-142-3p	STAT3, HIF-1α, PAD4	Regulate the methylation of pro-inflammatory/anti-inflammatory genes	TANs	MiR-223 ↓ → N2 polarization; MiR-142-3p ↑→ inhibits NETs and improves pro-inflammatory environment
lncRNA	MALAT1	EZH2	Recruit PRC2 to mediate H3K27me3	Tumour cells/DCs	Silencing of tumor suppressor genes promotes metastasis and immunosuppression
	HOTAIR	DNMT1/PRC2	Dual regulation of DNA methylation and H3K27me3	Tumour cells/DCs	Inhibit CD80/CD86 and induce immune tolerance
	NEAT1	EZH2/DNMT1	Stabilize the methylation complex	Tumour cells/T cells	Inhibit Th1 polarization and promote Treg amplification
	XIST	EZH2 → H3K27me3	Silence CDH1/KLF2	Tumour cells	Promote EMT and migration
	LINC01138	PRMT5	Enhance arginine methylation silencing	Tumour cells/TME	Induce an immunosuppressive phenotype
	SNHG16	HDAC1	Deacetylated p53 promoter	Tumour cells	Inhibit apoptosis and enhance immune tolerance
Exosome ncRNA	miR-21(from tumor → CAFs)	PI3K/Akt	Activate matrix remodeling and the secretion of growth factors	CAFs	Promote ECM generation, angiogenesis and tumor growth
	miR-101, miR-34a(→ T cells)	EZH2	Reduce H3K27me3 and relieve gene silencing	T cells	Restore antigen presentation and cytotoxic response
	HOTAIR(→ DCs)	DNMT1	Inhibit the expression of co-stimulatory factors	DCs	Induce immune tolerance
	NEAT1(→ T cells)	EZH2	Stabilize the methylation network	Treg/Th1	Inhibit Th1 polarization and promote Treg amplification
	miR-223/LINC00665(→ TANs)	STAT3/NF-κB	Activate immunosuppressive pathways	TANs	Promote N2 polarization and VEGF expression

### 4.4 Interaction network of epigenetic regulation in lung cancer microenvironment

**Figure 8:**
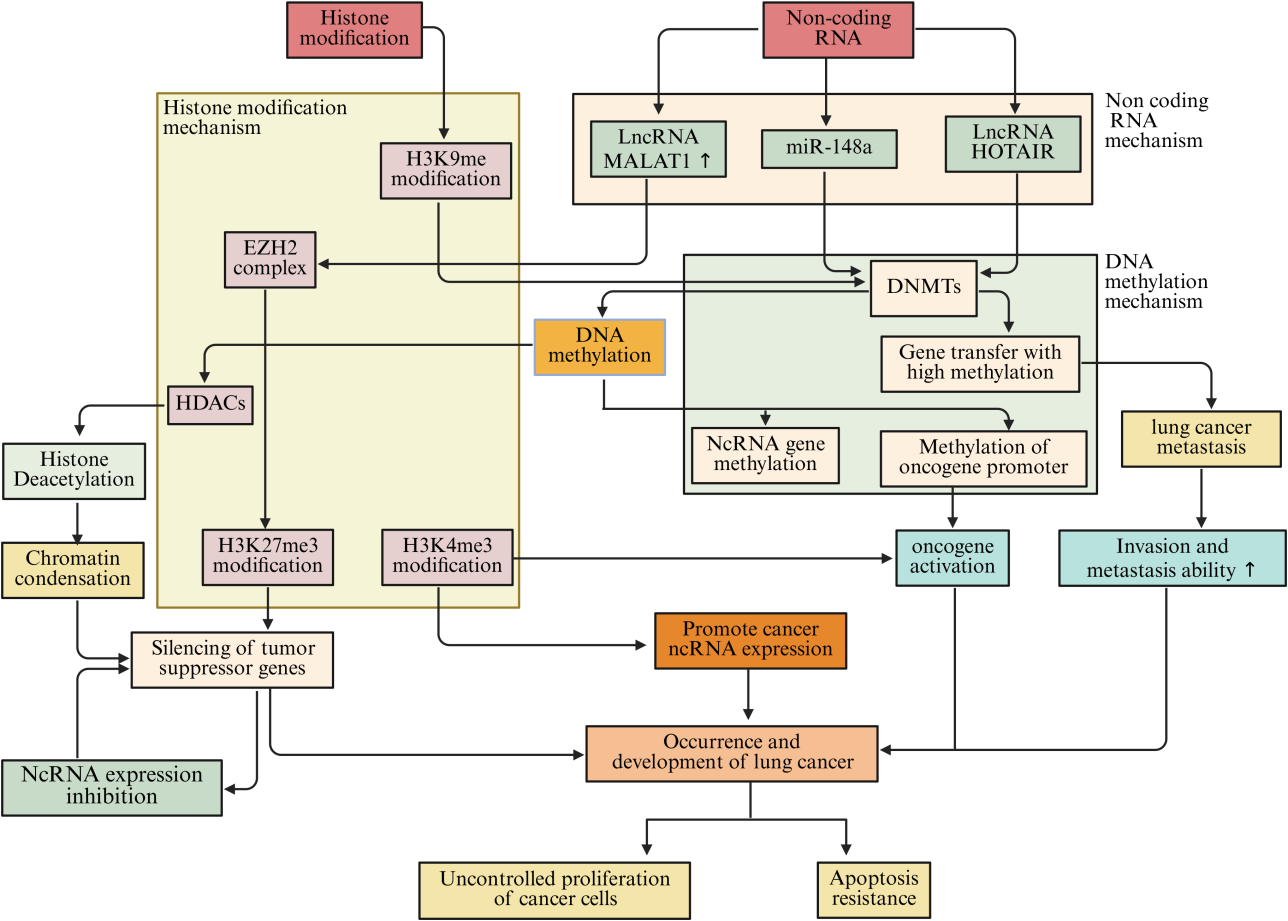
The interaction among DNA methylation, histone modification, and ncRNAs in the microenvironment of lung cancer jointly affects the biological behavior of lung cancer cells.

Epigenetic regulation in the microenvironment of lung cancer is a highly complex and delicate process. DNA methylation, histone modification, and ncRNAs do not act in isolation but interweave to form a tight regulatory network, jointly influencing the biological behavior of lung cancer cells as well as the communication and interaction between cells in the tumor microenvironment (**Figure 8**).

DNA methylation is closely related to histone modifications. DNA methylation can recruit protein complexes associated with histone modifications, thereby affecting the modification state of histones (253). In lung cancer cells, the DNA methylation binding protein MeCP2 can interact with HDACs and recruit them to DNA-methylated regions (254). HDACs remove acetyl groups from histones, which compacts the chromatin structure and inhibits gene transcription (255). Studies have found that in lung cancer, while certain tumor suppressor gene promoter regions are highly methylated, the acetylation levels of nearby histones decrease, leading to suppressed gene expression (256). This synergistic effect of DNA methylation and histone deacetylation silences tumor suppressor genes and promotes the initiation and progression of lung cancer (257). Conversely, histone modifications can also affect DNA methylation. Trimethylation of H3K9me3 can recruit DNMTs, promoting DNA methylation at target sites (258). In lung cancer, the H3K9me3 modification in some oncogene promoter regions increases, leading to elevated DNA methylation levels in those regions, upregulating oncogene expression, and driving the progression of lung cancer (259).

A reciprocal regulatory interaction exists between DNA methylation and ncRNAs. NcRNAs can influence DNA methylation through various mechanisms (260). MmiRNAs have the capacity to target and modulate the expression of DNMTs, thereby impacting the overall levels of DNA methylation (261). Studies indicate that miR-148a specifically targets DNMT1, leading to a suppression of its expression and a corresponding decrease in DNA methylation levels. In lung cancer cells, a reduction in miR-148a expression corresponds with an increase in DNMT1 levels, resulting in abnormal DNA methylation patterns, hypermethylation of specific tumor suppressor gene promoters, and subsequent gene silencing, which contributes to lung cancer progression (262). LncRNAs also play a significant role in the regulation of DNA methylation. Certain lncRNAs can interact with DNMTs, facilitating their recruitment to designated gene loci and enhancing DNA methylation processes (263). For instance, lncRNA HOTAIR has been shown to associate with DNMT1, DNMT3A, and DNMT3B, which are concentrated in the promoter regions of genes associated with tumor metastasis, resulting in hypermethylation of these genes and promoting the metastatic potential of lung cancer cells (264). On the other hand, DNA methylation can also affect the expression of non-coding RNA. Methylation of the promoter regions of some non-coding RNA genes can lead to their expression being suppressed (265). In lung cancer, hypermethylation of certain miRNA and lncRNA gene promoters results in downregulation of their expression, losing their normal regulatory effects on lung cancer cells, and affecting the progression of lung cancer (266).

Histone modifications also have complex interactions with non-coding RNAs. Histone modifications can alter the structure and accessibility of chromatin, affecting the transcription of non-coding RNA genes (267). For example, histone H3K4me3 modification is often associated with gene activation. In the promoter regions of some non-coding RNA genes, elevated levels of H3K4me3 modification can promote the transcription of these ncRNAs (268, 269). In lung cancer, some lncRNAs associated with tumor growth and metastasis show increased H3K4me3 modification in their gene promoter regions, leading to upregulation of lncRNA expression and contributing to tumor progression (270). Conversely, non-coding RNAs can also affect histone modifications. lncRNAs can interact with histone modification complexes to regulate histone modification patterns (271). MALAT1 can interact with the histone methyltransferase EZH2, promoting the methylation of histone H3K27 in the promoter regions of certain tumor suppressor genes, inhibiting the expression of these genes and promoting the invasion and metastasis of lung cancer cells (272). miRNAs can also indirectly influence histone modifications by regulating the expression of proteins involved in histone modification regulation. Specifically, miRNAs can target and inhibit some proteins involved in the regulation of histone modifications, thereby altering histone modification patterns. This affects gene expression and the biological behavior of lung cancer cells (273) (**Figure 8**).

## 5 Clinical perspective: challenges and opportunities

### 5.1 Challenges faced by epigenetic regulation of lung cancer microenvironment

From a clinical standpoint, the epigenetic modulation of the lung cancer microenvironment presents not only a myriad of challenges but also significant opportunities. These factors are intricately linked to the diagnosis, treatment, and prognostic evaluation of lung cancer patients, thereby exerting a considerable influence on the advancement of clinical interventions for this malignancy. Regarding clinical diagnosis, while epigenetic alterations within the lung cancer microenvironment have demonstrated potential as innovative biomarkers, several hurdles remain unaddressed. A predominant concern involves the constraints associated with existing detection methodologies. Current technologies for identifying DNA methylation, histone modifications, and ncRNAs exhibit limitations in sensitivity, specificity, operational simplicity, and affordability. For example, methylation-specific PCR (MSP) used in DNA methylation detection is susceptible to yielding both false positive and false negative results, and is capable of identifying only known methylation sites. Although bisulfite sequencing can offer comprehensive methylation insights across the entire genome, it comes with high costs, operational complexities, and necessitates substantial quantities of sample DNA. In the context of histone modification detection, the chromatin immunoprecipitation sequencing (ChIP-seq) method entails a cumbersome experimental protocol, demands high-quality antibodies, and involves sophisticated data analysis. The examination of ncRNAs also encounters challenges, particularly due to the brevity of miRNAs; standard RNA sequencing techniques struggle to accurately quantify their expression levels, and real-time quantitative PCR (qPCR) is also vulnerable to cross-reactivity when assessing miRNAs. Beyond these technical obstacles, there are additional complexities associated with the acquisition and processing of clinical samples. The cellular composition within the lung cancer microenvironment is highly intricate, with variations in the proportion and functional states of diverse cell types across patients, which may influence the detection outcomes of epigenetic markers. Furthermore, stringent conditions must be maintained throughout the collection, transportation, and storage of clinical specimens to preserve their quality and stability; any lapse in this process could lead to alterations in epigenetic modifications, ultimately compromising the accuracy of the tests.

In terms of treatment, the epigenetic regulation of the lung cancer microenvironment faces severe challenges as well. Developing drugs targeting epigenetic sites is an important direction, but there are still multiple challenges at various levels. The specificity and selectivity of epigenetic modification enzymes as drug targets remain core issues. For example, while DNA methyltransferase inhibitors (DNMT inhibitors) can reverse abnormal methylation states, they often also act on normal cells, leading to non-specific hypomethylation and off-target toxicity (274). Similarly, histone deacetylase inhibitors (HDAC inhibitors), while inhibiting tumor cell proliferation, can also disrupt the transcription balance of normal tissues, resulting in cardiovascular, hematopoietic, and nervous system side effects (275). Furthermore, epigenetic regulation has dynamic reversibility and complex feedback loops, allowing tumor cells to rapidly restore abnormal phenotypes through compensatory pathways (such as TET–DNMT balance, HAT-HDAC interaction, and ncRNA feedback loops), leading to acquired resistance (276, 277). Moreover, some epigenetic drugs (such as EZH2 inhibitors and BET inhibitors) have also shown an “early response—late rebound” phenomenon in clinical trials, indicating that the long-term efficacy and resistance control mechanisms remain unclear. In addition, the functional overlap of enzymatic activities among different epigenetic targets increases the complexity of combination therapy, making drug optimization and dosage control more challenging. On this basis, the efficacy evaluation of epigenetic drugs also faces bottlenecks. Due to their mechanism of action focusing on reshaping gene expression and immune status rather than directly inducing tumor shrinkage, traditional radiographic indices (such as the RECIST criteria) often fail to accurately reflect efficacy. The optimal assessment indicators for epigenetic regulatory drugs need to combine comprehensive parameters such as molecular-level methylation profiles, histone modification profiles, and immune cell functional outcomes, which poses higher requirements for clinical monitoring systems.

From the perspective of clinical translation, converting research findings on the epigenetic regulation of the lung cancer microenvironment into effective therapeutic methods remains a major challenge. Currently, most studies are at the basic and preclinical stages, and although positive results have been achieved in cell lines and animal models, efficacy in clinical trials is often inconsistent. For instance, DNMT inhibitors can restore tumor suppressor gene expression and inhibit tumor growth *in vitro*, but in clinical settings, they often show limited efficacy and serious toxic side effects, such as bone marrow suppression (278). This discrepancy partly arises from the complex immune-metabolic-epigenetic interaction network within the tumor microenvironment. This network causes drug effects to be influenced by individual differences and microenvironmental plasticity, which refers to the dynamic and adaptable nature of the tumor microenvironment. Additionally, the high heterogeneity of epigenetic characteristics among lung cancer patients leads to significant differences in individual responses to the same epigenetic drugs. Therefore, establishing precision medicine strategies based on epigenetic profiling and developing individualized drug sensitivity prediction models will be key directions for the successful translation of epigenetic therapies for lung cancer in the future.

### 5.2 Opportunities brought by epigenetic regulation of lung cancer microenvironment

Epigenetic regulation of the microenvironment in lung cancer has also brought unprecedented opportunities for clinical treatment. These opportunities mainly lie in the epigenetic modifications in the tumor microenvironment, which act as novel biomarkers. They provide new approaches for the early diagnosis and prognostic assessment of the disease, thereby offering strong support for new targets and strategies in its treatment.

#### 5.2.1 Provide new markers for the diagnosis and prognosis assessment of lung cancer

The timely identification and precise prognostic evaluation of lung cancer are essential for enhancing treatment efficacy and improving patient survival rates. Conventional diagnostic approaches primarily depend on imaging techniques—such as chest radiographs and computed tomography scans—alongside histopathological assessments and tumor marker analysis. However, these techniques exhibit several limitations. For example, imaging modalities often demonstrate insufficient sensitivity for the early detection of lung cancer, while histopathological evaluations are invasive and may be prone to sampling inaccuracies. Furthermore, the specificity and sensitivity of standard tumor markers, including carcinoembryonic antigen (CEA) and cytokeratin 19 fragment (CYFRA21-1), require further refinement for effective diagnosis and prognostic evaluation of lung cancer. Recently, epigenetic alterations within the lung cancer microenvironment have emerged as promising novel biomarkers, offering innovative strategies for the diagnosis and prognostic evaluation of this malignancy.

DNA methylation, as an important epigenetic modification, exhibits characteristic changes during the occurrence and development of lung cancer, making it a potential biomarker for lung cancer diagnosis and prognosis assessment (279). Studies have found that abnormal methylation of various genes is closely related to the occurrence of lung cancer. These genes include tumor suppressor genes, oncogenes, and those involved in tumor metabolism, invasion, and metastasis. The RASSF1A gene is an important tumor suppressor gene whose promoter region frequently undergoes hypermethylation in lung cancer tissues, leading to gene silencing. Detecting the methylation status of the RASSF1A gene promoter can serve as a potential biomarker for lung cancer diagnosis (280). Similarly, methylation of the APC gene is also associated with lung cancer occurrence, with hypermethylation of the APC gene promoter detectable in both lung cancer tissues and patient plasma. Monitoring the methylation status of the APC gene aids in the early diagnosis of lung cancer, especially for early lesions, which are difficult to detect through imaging examinations (281). In addition to diagnosis, DNA methylation markers can also be used for prognosis assessment in lung cancer. Hypermethylation of the DAPK1 gene promoter is associated with poor prognosis in lung cancer patients, with methylation-positive patients-defined as those exhibiting promoter hypermethylation-having significantly shorter progression-free survival and overall survival compared to methylation-negative patients (282, 283). By detecting the methylation status of the DAPK1 gene, a better assessment of patient prognosis can be achieved, providing a basis for developing personalized treatment plans. In addition, the DNA methylation profile not only reflects the molecular typing characteristics of tumors but also provides important evidence for patient stratification. By integrating the methylation status of multiple genes (such as RASSF1A, p16, CDH1, and TMEFF2), researchers can distinguish between the “highly methylated phenotype” (CIMP) and the “low methylation phenotype” subgroups (284). The CIMP-positive subtype is usually associated with an immunosuppressive microenvironment, high expression of PD-L1, and sensitivity to epigenetic drugs, while CIMP-negative patients often exhibit immune inflammatory characteristics and respond better to immunotherapy. Therefore, detection of methylation characteristics can be used not only for early diagnosis and prognosis evaluation but also to lay a molecular foundation for personalized therapy and stratified management of lung cancer patients.

Histone modifications also undergo abnormal changes in the lung cancer microenvironment, which are closely related to the biological behaviors and prognosis of lung cancer, and can serve as potential biomarkers for the diagnosis and prognosis assessment of lung cancer. The trimethylation of H3K9me3 is associated with gene silencing. In lung cancer cells, the levels of H3K9me3 in the promoter regions of certain tumor suppressor genes are elevated, leading to gene silencing (285). Studies have found that the overall level of H3K9me3 in lung cancer tissues is related to the staging and grading of tumors, as well as the prognosis of patients (286). In early-stage lung cancer patients, the levels of H3K9me3 in tumor tissues are lower, while in late-stage lung cancer patients, the levels of H3K9me3 are significantly elevated. Detection of H3K9me3 levels in lung cancer tissues aids in assessing tumor progression and patient prognosis (287). Trimethylation of H3K4me3, is usually associated with gene activation. In lung cancer, the levels of H3K4me3 in the promoter regions of some genes related to tumor growth and metastasis are elevated. By detecting the levels of H3K4me3 in these gene promoter regions, the proliferation and metastatic ability of lung cancer cells can be assessed, providing a reference for the prognosis evaluation of lung cancer (288). Furthermore, different combinations of histone modifications constitute the “epigenetic subtypes” of lung cancer. For example, high levels of H3K27me3 caused by EZH2 overactivation often indicate an immunologically cold subtype, which is less responsive to ICIs but sensitive to EZH2 inhibitors; the loss of H3K36me3 caused by SETD2 mutations is associated with an immunologically hot phenotype (289). In the future, a multidimensional stratification model combining histone modification characteristics is expected to achieve precise matching of immune therapies and epigenetic drug combination predictions for lung cancer patients.

NcRNAs, especially miRNAs and lncRNAs, are abnormally expressed in the lung cancer microenvironment and are closely related to the occurrence, development, metastasis, and prognosis of lung cancer, showing great potential as biomarkers for lung cancer diagnosis and prognosis assessment (290). miR-21 is highly expressed in lung cancer tissues and cell lines, and its expression level is associated with the staging, grading, and prognosis of lung cancer. Studies have found that the level of miR-21 in the serum of lung cancer patients is significantly higher than that of healthy controls, and lung cancer patients with high expression of miR-21 have a poorer prognosis. Detecting the level of miR-21 in serum can assist in the diagnosis of lung cancer and assess the prognosis of patients (291, 292). miR-155 is also highly expressed in the lung cancer microenvironment, and it is involved in regulating the proliferation, invasion, and metastasis of lung cancer cells. Clinical studies have shown that the expression level of miR-155 is closely related to the survival of lung cancer patients, with patients expressing high levels of miR-155 having significantly shorter survival times (293). Furthermore, detecting the expression level of miR-155 can serve as an important indicator for lung cancer prognosis assessment. Research on lncRNAs in lung cancer is also receiving increasing attention, with many lncRNAs such as MALAT1 and HOTAIR being abnormally expressed in lung cancer tissues. The high expression of MALAT1 is associated with metastasis and poor prognosis in lung cancer. By detecting the expression level of MALAT1 in lung cancer tissues or patient plasma, the metastatic potential of lung cancer and the prognosis of patients can be assessed (294). NcRNAs, especially circulating miRNAs and exosomal lncRNAs, provide new avenues for dynamic stratification of patients during treatment. The expression profiles of different ncRNA combinations can reflect tumor immune status and treatment sensitivity; for example, the high expression subtype of miR-21/miR-155 usually corresponds to immunosuppressive lung cancer, while low expression of miR-126 and the miR-200 family suggests immune-active characteristics (295). In addition, exosomal ncRNAs as liquid biopsy epigenetic biomarkers can be used for dynamic monitoring of treatment response and resistance evolution. By integrating miRNA, lncRNA, and DNA methylation signals to construct the “Epigenetic–Immune Signature Score,” a comprehensive and precise assessment from diagnosis and stratification to efficacy prediction can be achieved (296, 297).

#### 5.2.2 Provide new targets and strategies for the treatment of lung cancer

Comprehensive investigations into the epigenetic regulatory mechanisms within the lung cancer microenvironment have unveiled novel therapeutic targets and strategies, potentially enhancing treatment efficacy and prognostic outcomes for lung cancer patients. Among these, the therapeutic approaches related to DNA methylation have emerged as significant focal points in research. DNMTs represent critical enzymes that facilitate the process of DNA methylation. In the context of lung cancer, the aberrant expression of DNMTs results in altered DNA methylation patterns, characterized by hypermethylation occurring in the promoter regions of numerous tumor suppressor genes, which ultimately leads to the silencing of gene expression. Consequently, DNMTs have been identified as promising therapeutic targets, prompting the development of DNMTis aimed at lung cancer treatment.

The earliest DNMTis investigated include 5-aza-2-deoxycytidine (5-aza-dC) and 5-aza-cytidine (5-aza-CR). These agents can be incorporated into DNA, forming covalent bonds with DNMTs, thereby inhibiting their enzymatic activity and leading to a reduction in DNA methylation levels. This mechanism facilitates the reactivation of tumor suppressor gene expression (298, 299). Experimental studies conducted on lung cancer cell lines and animal models have demonstrated that both 5-aza-dC and 5-aza-CR effectively inhibit lung cancer cell proliferation, induce apoptosis, and suppress tumor growth and metastatic spread (300). Nevertheless, it is important to note that despite these beneficial effects, these drugs exhibit certain limitations, including high cytotoxicity and a lack of specificity. Recently, novel DNMTis such as decitabine and zebularine have been developed. Decitabine is a modified DNMTi that possesses enhanced activity and reduced toxicity, showing promising therapeutic potential for lung cancer in clinical trials (301). Zebularine has better cell permeability and stability and can more effectively inhibit the activity of DNMTs (302). In addition to directly inhibiting the activity of DNMTs, zebularine can also affect the DNA methylation status by regulating the signaling pathways related to DNA methylation (303). Research has identified several signaling pathways, including the Wnt/β-catenin and PI3K/Akt pathways, as being intimately linked to DNA methylation processes. By targeting and inhibiting these pathways, it is possible to indirectly regulate DNMT activity and DNA methylation levels, presenting a novel therapeutic strategy for lung cancer management (304).

Therapeutic targets and strategies related to histone modifications have also made significant progress. HDACs are key enzymes that regulate histone acetylation levels. In lung cancer, the activity of HDACs is elevated, leading to enhanced histone deacetylation and suppression of tumor suppressor gene expression (305). Therefore, HDAC inhibitors (HDACi) have become potential drugs for lung cancer treatment. Vorinostat, Romidepsin, and Belinostat are commonly used HDAC inhibitors in clinical practice. These drugs inhibit the activity of HDACs, increase histone acetylation levels, and reactivate the expression of tumor suppressor genes, thereby inhibiting the growth and proliferation of lung cancer cells (306). In lung cancer cell lines and animal models, HDACi can induce apoptosis of lung cancer cells, inhibit cell cycle progression, and suppress tumor metastasis to some extent. Some HDACi have entered clinical trials, bringing new hope to lung cancer patients (307, 308). In addition to HDAC inhibitors, inhibitors targeting HMTs are also under investigation. EZH2 is an important HMT that catalyzes the trimethylation of H3K27me3. In lung cancer, the expression of EZH2 is often upregulated, leading to elevated levels of H3K27me3 and silencing of many tumor suppressor genes (309). Inhibitors targeting EZH2, such as EPZ-6438, and GSK126, can reduce H3K27me3 levels, reactivate tumor suppressor genes, and inhibit the growth and metastasis of lung cancer cells. These inhibitors have shown good efficacy in preclinical studies and are expected to become new drugs for lung cancer treatment (310).

Therapeutic targets and strategies related to non-coding RNA have opened new directions for lung cancer treatment. As a type of ncRNA, miRNAs are abnormally expressed in lung cancer and are closely related to its occurrence and development (311). Regulating the expression or activity of miRNAs can influence the biological behavior of lung cancer cells, thereby offering new approaches for lung cancer therapy. Antisense oligonucleotides, including chemically modified antagomiRs, can be designed to inhibit the function of oncogenic miRNAs that are highly expressed (312). For oncogenic miRNAs such as miR-21, which is highly expressed in lung cancer, antagomiR-21 can specifically bind to miR-21 and inhibit its regulatory effect on target genes; this suppresses the proliferation, invasion, and metastasis of lung cancer cells (313). Research has also found that some miRNAs can act as tumor suppressors, and restoring their expression can contribute to lung cancer treatment. For example, miR-34a is downregulated in lung cancer, and introducing miR-34a mimics into lung cancer cells via gene delivery technology can inhibit tumor growth and metastasis while inducing apoptosis (314, 315). The role of lncRNA in lung cancer is also receiving increasing attention. For certain lncRNAs closely related to lung cancer progression, such as MALAT1 and HOTAIR, small interfering RNA (siRNA) or short hairpin RNA (shRNA) can be designed to inhibit their expression, thereby affecting the biological behavior of cancer cells (316). Specifically, inhibiting MALAT1 expression reduces the migration and invasion abilities of lung cancer cells, suppressing tumor metastasis (317). Furthermore, developing small molecule inhibitors or antibodies that target the interactions between lncRNAs and other molecules represents another promising research direction for lung cancer treatment (318).

Combining epigenetic therapy with traditional treatment methods, to achieve combination therapy, is an important strategy to improve the efficacy of lung cancer treatment. Epigenetic therapy can enhance the sensitivity of tumor cells to traditional treatment methods such as chemotherapy, radiotherapy, targeted therapy, and immunotherapy by altering the epigenetic modifications of tumor cells (319). In chemotherapy, DNMTi and HDACi can increase the sensitivity of lung cancer cells to chemotherapeutic agents by reactivating tumor suppressor genes. Studies have found that in lung cancer cell lines, treatment with 5-aza-2’-deoxycytidine (5-aza-dC) in combination with cisplatin is more effective than cisplatin alone. This results in more significant inhibition of lung cancer cell proliferation and increased apoptosis (320). Similarly, in radiotherapy, epigenetic therapy can improve the effectiveness of radiotherapy by regulating the DNA damage repair mechanisms and the immune microenvironment of tumor cells. HDACi can increase the sensitivity of tumor cells to radiotherapy. Additionally, they modulate the function of immune cells in the tumor microenvironment, enhancing the body’s anti-tumor immune response (321). In targeted therapy, epigenetic therapy can overcome the drug resistance of tumor cells. For lung cancer cells resistant to epidermal growth factor receptor (EGFR) tyrosine kinase inhibitors (TKI), the use of DNMTi or HDACi can alter the epigenetic modifications of drug resistance-related genes, partially restoring the sensitivity of lung cancer cells to TKI (306, 322). In immunotherapy, epigenetic therapy can enhance the efficacy of immunotherapy by regulating the function of immune cells and the immune signaling pathways in the tumor microenvironment (323). In recent years, the widespread application of immune checkpoint inhibitors (such as PD-1/PD-L1 and CTLA-4 antibodies) has made the synergistic potential of epigenetic therapy and immunotherapy a research hotspot. Tumor immune evasion is often accompanied by multi-layered epigenetic reprogramming, including DNA methylation, histone deacetylation, and abnormal expression of non-coding RNAs. These changes lead to defects in antigen presentation and increased infiltration of immunosuppressive cells (324). Epigenetic therapy can reverse these changes and activate previously silenced immune pathways, thereby enhancing immunotherapy. Specifically, DNMTi and HDACi can relieve the methylation silencing of tumor-associated antigens (TAAs) and genes of antigen processing complexes (MHC-I, TAP1/2). This restoration improves antigen presentation capability (324). At the same time, DNMTi can induce a viral mimicry response by activating the transcription of endogenous retroviruses and upregulating interferon signaling, which enhances immunogenicity (325). Epigenetic drugs can also improve the function of immune effector cells. For example, HDACi promotes the memory differentiation of CD8^+^T cells and inhibits the expression of exhaustion markers. BET inhibitors (BETi) suppress the M2 polarization of TAMs. EZH2 inhibitors (EZH2i) weaken the immunosuppressive effects of Tregs (325). Moreover, in “cold-type” lung cancer, epigenetic regulators can activate chemokines such as CXCL9 and CXCL10, transforming it into a “hot-type” tumor and significantly enhancing the response rate to PD-1/PD-L1 inhibitors. Preclinical studies have demonstrated significant synergistic effects when combining DNMTi with PD-1 inhibitors, HDACi with PD-L1 inhibitors, and EZH2i with CTLA-4 inhibitors. A stratified treatment strategy based on epigenomic characteristics (such as CIMP status, EZH2 expression levels, and Treg enrichment) is expected to enhance the precision and clinical translation of epigenetic-immunotherapy combination therapies (326, 327).

## 6 Future research directions and prospects

The investigation into the epigenetic regulation of the tumor microenvironment in lung cancer presents extensive potential and considerable significance. In the realm of technological advancements, the imperative lies in creating more sophisticated and sensitive methodologies that facilitate single-cell and real-time dynamic detection. Additionally, enhancing single-cell sequencing techniques to improve their throughput and precision will permit a more thorough examination of the epigenetic traits exhibited by various cell types within the tumor microenvironment. The innovation of new detection platforms leveraging nanotechnology, microfluidics, and other methodologies is vital to achieve highly sensitive identification of low-abundance epigenetic markers in the lung cancer microenvironment while also enabling real-time observation of the temporal dynamics of epigenetic alterations. Technologies that amalgamate nanosensors with microfluidic devices are capable of detecting, in real time, fluctuations in DNA methylation markers released by tumor cells into the bloodstream of lung cancer patients throughout treatment. This capability serves as a foundation for timely modifications of treatment strategies.

Regarding mechanistic exploration, it is essential to thoroughly investigate the intricate network of epigenetic regulation within the lung cancer microenvironment and its dynamic alteration patterns. By employing systems biology and bioinformatics methodologies and integrating multi-omics data, a more comprehensive model of the epigenetic regulatory network pertaining to the lung cancer microenvironment can be established. This model will facilitate a holistic analysis of interactions between distinct cell types, diverse epigenetic modifications, and the interplay of epigenetic regulation with signaling pathways and cellular components. Furthermore, it is crucial to conduct a detailed examination of the temporal dynamics of epigenetic regulatory mechanisms within the tumor microenvironment across various developmental stages and treatment phases, thus providing a theoretical framework for the comprehensive management of lung cancer. As lung cancer progresses from early to advanced stages, analyzing the temporal dynamics of epigenetic changes in tumor cells and immune cells at various time intervals, along with assessing the influence of these changes on tumor immune evasion and metastasis, is essential. Such investigations lay the groundwork for the formulation of targeted treatment strategies.

The primary objective of research concerning epigenetic regulation in the lung cancer microenvironment is its clinical translation. Moving forward, it is imperative to enhance the synergy between fundamental research and clinical application. Researchers ought to undertake extensive, multi-center clinical trials to validate the effectiveness and safety of diagnostic biomarkers and therapeutic targets influenced by epigenetic mechanisms. It is essential to establish a comprehensive epigenetic database for lung cancer patients, which should encompass their clinical data and treatment responses. This database will facilitate the development of precise, individualized treatment prediction models aimed at achieving accurate diagnosis and management of lung cancer. Additionally, there is a need to further refine the integrative treatment approaches that combine epigenetic therapies with conventional treatment modalities, investigate novel combinatorial treatment frameworks, and enhance the therapeutic outcomes for lung cancer patients. Furthermore, it is crucial to examine the optimal dosing regimens and administration sequences of DNMTi in conjunction with immune checkpoint inhibitors for lung cancer treatment. This should be complemented by preclinical and clinical trials to assess the safety and efficacy of such combination therapies, ultimately offering more effective treatment alternatives for lung cancer patients.

Despite the numerous challenges associated with investigating the epigenetic regulation of the lung cancer microenvironment, there are significant opportunities for advancement. Through ongoing technological innovations, comprehensive mechanistic studies, and efforts toward clinical translation, it is anticipated that novel breakthroughs will emerge in the prevention and treatment of lung cancer, thereby substantially enhancing the prognosis and quality of life for individuals affected by this disease.

## 7 Conclusion

Lung cancer represents a malignant neoplasm that poses a significant threat to global health and exhibits a multifaceted etiology. Nonetheless, there remains a critical need to enhance both the therapeutic effectiveness and prognostic outcomes for affected individuals. While investigations into the epigenetic modulation of the lung cancer microenvironment encounter considerable obstacles, they simultaneously present promising avenues for progress. Several novel biomarkers have been discovered for the diagnosis and prognosis of lung cancer, which encompass DNA methylation indicators, histone modification markers, and ncRNA signatures. These biomarkers aid in facilitating earlier detection and more precise prognostic evaluations. Additionally, innovative therapeutic targets and strategies have emerged, including interventions that focus on DNA methylation, histone modifications, and ncRNA pathways. The implementation of combination treatment regimens that merge epigenetic therapies with conventional approaches is anticipated to improve therapeutic outcomes and patient prognoses. Furthermore, investigations into epigenetic regulation have catalyzed advancements in precision medicine for lung cancer. By assessing the unique epigenetic profiles of patients, tailored treatment plans can be formulated, thereby enhancing both the safety and efficacy of therapeutic interventions. In summary, research focused on epigenetic regulation within the lung cancer microenvironment offers a crucial pathway for elucidating the disease’s underlying mechanisms and pioneering innovative treatment methodologies.
